# Prognostic value of micro-RNA in ovarian cancer: a systematic review and meta-analysis

**DOI:** 10.3389/fonc.2025.1641602

**Published:** 2026-01-12

**Authors:** Zhenzhen He, Lihong Deng, Zhipeng Wang

**Affiliations:** 1 Gynaecology, Tianjin First Central Hospital, Nankai University, Tianjin, China; 2 Institute of Preventive Medicine, Tianjin Centers for Disease Control and Prevention, Tianjin, China; 3 Institute of Transplantation Medicine, Tianjin First Central Hospital, Nankai University, Tianjin, China

**Keywords:** hazard ratio, meta-analysis, microRNA, ovarian cancer, prognosis

## Abstract

**Background:**

Previous studies investigating the association between microRNAs (miRNAs) and ovarian cancer prognosis have yielded inconsistent results. This study aims to synthesize all available evidence through a systematic review and meta-analysis to provide a comprehensive assessment of the prognostic value of miRNAs in ovarian cancer.

**Methods:**

A systematic search was conducted in PubMed (from 1965), ISI Web of Science (from 1900), MEDLINE (from 1976), and Scopus (from 1968) through September 9, 2024. Studies published in English, examining the relationship between miRNAs and ovarian cancer prognosis, were included. miRNAs were categorized as upregulated or downregulated based on their expression levels in ovarian cancer tissues compared to normal tissues. The primary outcomes were the hazard ratios (HRs) for overall survival (OS) and progression-free survival (PFS) of ovarian cancer patients.

**Results:**

A total of 51 studies involving 6916 ovarian cancer patients and 181 distinct miRNAs were included in the meta-analysis. Upregulated miRNAs were significantly associated with better OS in Chinese ovarian cancer patients compared to patients from other regions (HR 0.43 [95% CI: 0.20-0.65] vs. 1.01 [95% CI: 0.90-1.11], P<0.01). For serous carcinoma, the HR for upregulated miRNAs related to OS was 0.51 (95% CI: 0.11-0.92). Specifically, elevated expression of the miR-200 family was significantly associated with improved OS (HR 0.66 [95% CI: 0.53-0.80]) and PFS (HR 0.68 [95% CI: 0.47-0.89]) in ovarian cancer. Similarly, higher expression of the miR-30 family correlated with improved OS (HR 0.63 [95% CI: 0.39-0.87]) and PFS (HR 0.70 [95% CI: 0.54-0.87]).

**Conclusion:**

Up-regulated miRNAs show prognostic value in Chinese patients; however, regional influences warrant further investigation. Specifically, miRNAs may serve as important prognostic indicators for serous carcinoma. Different miRNA families, particularly miR-200 and miR-30, have the potential to act as key biomarkers for ovarian cancer prognosis.

**Systematic Review Registration:**

https://www.crd.york.ac.uk/PROSPERO/view/CRD42024579585, identifier CRD42024579585.

## 1 Introduction

In 2024, an estimated 20 thousand new cases of ovarian cancer and 13 thousand deaths from ovarian cancer occurred in the United States (1). Although improvements in treatment methods have increased the survival rates of ovarian cancer, it remains one of the most lethal gynecological malignancies, with a high mortality rate due to late-stage diagnosis and the lack of effective early detection methods (2). The current standard of care for ovarian cancer is primarily guided by disease stage and histology, involving cytoreductive (debulking surgery) for complete tumor resection (R0), followed by first-line platinum-based chemotherapy with paclitaxel, with adjuvant targeted therapy and radiotherapy applied based on genetic profiles and clinical needs (3). Despite advances in surgical techniques and chemotherapy, the overall survival for patients with advanced ovarian cancer remains low. Prognosis prediction in ovarian cancer is complex, relying on a combination of clinical parameters, histopathological features, and molecular biomarkers. In recent years, microRNAs (miRNAs) have emerged as important regulators of gene expression and have shown promise as potential biomarkers for cancer prognosis (4).

MicroRNAs are small, non-coding RNAs that play crucial roles in regulating various cellular processes, including cell proliferation, apoptosis, differentiation, and invasion (5). In cancer, the aberrant expression of specific miRNAs is associated with tumorigenesis, progression, and metastasis. Furthermore, miRNAs are stable in body fluids and tumor tissues, making them attractive candidates for non-invasive biomarkers in cancer diagnosis and prognosis (6).

Several studies have suggested that specific miRNAs may serve as prognostic biomarkers for ovarian cancer, influencing key aspects of tumor behavior such as drug resistance, recurrence, and metastasis. However, the results from individual studies have been inconsistent, likely due to variations in sample sources, sample size, assay methods used for miRNA detection, tumor characteristics and patient populations. For instance, the hazard ratio (HR) of miRNA-200c associated with the overall survival of ovarian cancer ranged from 0.06 (95%CI: 0.03-0.79) (7) to 2.40 (95%CI: 1.20-4.90) (8). Similar results have been observed in other type of miRNA, such as miRNA-145 with an HR of overall survival ranging from 0.12 (9) to 16.67 (10). To address these discrepancies and provide a more comprehensive understanding of the role of miRNAs in ovarian cancer prognosis, a meta-analysis is needed to systematically evaluate the association between miRNA expression levels and clinical outcomes in ovarian cancer patients. In addition, due to the heterogeneous nature of ovarian cancer, several histological subtypes have been identified, including serous, clear cell, endometrioid, and mucinous. These different histotypes even represent distinct diseases at the clinical, morphological, immunohistochemical, and genetic levels (11). Therefore, after exploring the association between miRNAs and overall ovarian cancer prognosis, further investigation of the relationship between miRNAs and prognosis in different ovarian cancer subtypes, based on histological classification, will help to gain a deeper understanding of the role of miRNAs in ovarian cancer prognosis. This comprehensive analysis could not only provide potential prognostic biomarkers but also identify specific miRNAs that may serve as therapeutic targets for precision treatment of different subtypes. By elucidating the distinct miRNA profiles associated with different ovarian cancer histotypes and their relationship to patient prognosis, this work may reveal novel molecular pathways that could be exploited for targeted therapies. Previous meta-analyses exploring the association between miRNAs and ovarian cancer prognosis have been either based on a single database (12) or focused on specific miRNAs (13, 14). Thus, this study aims to integrate all the available evidence and provide a more comprehensive assessment of the prognostic value of miRNAs in ovarian cancer, ultimately contributing to improved patient stratification and personalized treatment approaches.

## 2 Materials and methods

### 2.1 Search strategy and selection criteria

This systematic review and meta-analysis were conducted in accordance with the Preferred Reporting Items for Systematic Reviews and Meta-Analyses (PRISMA) guidelines (see **Supplementary Table S1**) and are registered on the International Prospective Register of Systematic Reviews (registration number: CRD42024579585) (15). A comprehensive literature search was performed across four electronic English-language databases: PubMed (National Library of Medicine, Bethesda, MD), ISI Web of Science (Thomson Reuters, New York, NY), MEDLINE (via EBSCOhost), and Scopus. The following search terms, along with their corresponding MeSH terms, were used: “ovarian cancer,” “microRNA,” and “prognosis” (see **Supplementary Table S2**). Additionally, we examined the reference lists of relevant articles to identify further studies. Publications up to September 9, 2024, were included in the search. The starting years for the databases were as follows: PubMed (1965), Web of Science (1900), MEDLINE (1976), and Scopus (1968). The inclusion criteria were: (1) the study must be longitudinal, (2) the study must report on at least one type of miRNA, and (3) the study must provide prognostic outcomes for ovarian cancer, such as overall survival (OS) or progression-free survival (PFS). Studies were excluded if: (1) they were not published in English, (2) they were reviews, systematic reviews, conference abstracts, or case reports, or (3) hazard ratios (HRs) and 95% confidence intervals (CIs) could not be extracted or estimated from the article. As this study is a systematic review and meta-analysis based on publicly available data, and does not involve new human trials or private data, ethical committee approval was not required.

### 2.2 Data extraction and quality evaluation

Article eligibility was assessed independently by two authors (ZH and LD), who also handled data extraction and quality evaluation of the eligible studies. Discrepancies were resolved with the help of a third person (ZW) to reach a consensus. Agreement between the authors on study selection and data extraction was measured using the agreement rate and kappa coefficient. Extracted data included the first author’s name, publication year, study design, region, type of miRNAs, test methods used for miRNA detection, number of patients, mean or median age, and study sample source. The methodological quality of the studies was evaluated using the Newcastle-Ottawa Scale (**Table 1**), which assesses three domains: selection (four items), comparability (one item), and outcome (three items) (16).

**Table 1:** Characteristics of the included studies.

Study	MicroRNA	Population	Sample size	Assay	Cut-off value	FIGO stage (percentage of III-IV)	Histotypes (percentage of serous)	Sample	Outcome	Age	NOS score
Calura (2016) (9)	let-7a	Italy	157	qRT-PCR	ROC	–	27.4%; high grade serous 21%	tissue	OS/PFS	mean 53 (range 16–81)	9
Lu (2011) (55)	let-7a	Italy	211	qRT-PCR	tertile	75%	43%	tissue	OS/PFS	mean 57 (range 26–82)	9
Zheng (2013) (18)	let-7f	China	360	qRT-PCR	–	63%	50%	plasma	OS/PFS	mean 53	9
Peng (2012) (19)	miR-100	China	98	qRT-PCR	median	56%	28%	tissue	OS	<50y 34; ≥50y 64	9
Minareci (2024) (62)	miR-1181	Turkey	69	qRT-PCR	ROC	72%	75%	serum	OS/PFS	mean 61 (range 23–80)	9
Prahm (2018) (48)	miR-1183	Denmark	197	qRT-PCR	–	73%	82%	FFPE	OS/PFS	median 64 (range 31–89)	9
Flores-Colón (2024) (43)	miR-1206	KM plotter database (www.kmplot.com, accessed on 20 August 2023)	486	NanoString	median	–	–	tissue	OS	–	9
Xie (2020) (20)	miR-1231	China	116	qRT-PCR	–	41%	–	tissue	OS	≤50y 53; >50y 63	8
Liu (2023) (21)	miR-126	China	69	qRT-PCR	median	52%	72%	tissue	OS/PFS	<45y 25; ≥45y 44	9
Guo (2018) (22)	miR-1294	China	69	qRT-PCR	–	57%	–	tissue	OS	–	8
Bagnoli (2016) (3)	miR-141	Italy	179	qRT-PCR	median	82%	69%	tissue	OS	median 58 (range 28–78)	9
Kim (2015) (8)	miR-145	Korea	74	qRT-PCR	low: <0.04; high: >0.04	72%	high grade serous 100%	FFPE	OS	median 58	9
Gong (2016) (23)	miR-148a	China	102	qRT-PCR	mean	41%	–	plasma	OS	mean 52.23 (range 32–75)	9
Panoutsopoulou (2020) (50)	miR-181a	Germany	81	qRT-PCR	X-tile algorithm	–	–	tissue	OS/PFS	median 62 (range 25-82)	9
Chen (2016) (24)	miR-183	China	75	qRT-PCR	mean	57%	25%	tissue	OS	<50y 55; ≥50y 20	8
Qin (2015) (25)	miR-184	China	80	qRT-PCR	–	70%	–	tissue	OS	<55y 35; ≥55y 45	8
Li (2015) (26)	miR-193b	China	116	qRT-PCR	median	46%	48%	tissue	OS	≤50y 54; >50y 62	9
Fan (2015) (27)	miR-196a	China	156	qRT-PCR	median	54%	50%	tissue	OS/PFS	≤50y 57; >50y 89	9
Petrillo (2016) (56)	miR-199	Italy	82	qRT-PCR	–	100%	87%	FFPE	OS/PFS	mean 62.5 (range 31–83)	8
Meng (2016) (7)	miR-200	Germany	163	qRT-PCR	median	44%	60%	exosome	OS/PFS	mean 60 (range 23–91)	9
Gao (2015) (28)	miR-200	China	93	qRT-PCR	–	22%	17%	serum	OS	–	7
Cao Q (2014) (6)	miR-200	China	100	qRT-PCR	median	100%	75%	tissue	OS	median 58	9
Leskelä (2011) (61)	miR-200	Spain	72	ISH	expression level	79%	80%	FFPE	OS/PFS	median 57 (range 35-85)	9
Hu (2009) (63)	miR-200	USA	55	qRT-PCR	10.8	72%	74%	tissue	OS/PFS	–	8
Kapetanakis (2015) (49)	miR-200b	France	51	qRT-PCR	0	–	–	plasma	PFS	mean 62 (range 32-81)	9
Marchini (2011) (57)	miR-200c	Italy	89	qRT-PCR	Contal and O’Quigley method	100%	high grade serous 100%	tissue	OS/PFS	median 52 (IQR 21–82)	9
Elgaaen (2014) (60)	miR-200c	Norway	35	qRT-PCR	tertile	–	33%	tissue	OS/PFS	mean 64 (range 45–87)	9
Panoutsopoulou (2020) (51)	miR-203	Germany	103	qRT-PCR	X-tile algorithm	84%	79%; high grade serous 65%	tissue	OS/PFS	median 62 (range 25-83)	9
Yu (2020) (29)	miR-206	China	316	qRT-PCR	median	88%	100%; high grade serous 90%	FFPE	OS	–	8
Pan (2016) (30)	miR-217	China	129	qRT-PCR	median	67%	63%	tissue	OS	≤65y 55; >65y 74	8
Wan (2014) (31)	miR-22	China	109	qRT-PCR	median	44%	65%	tissue	OS	<55y 46; ≥55y 63	9
Fu (2016) (32)	miR-222	China	74	qRT-PCR	low: <1.6; high: >1.6	55%	46%	FFPE	OS	median 49	8
Pal (2024) (53)	miR-27a	India	208	qRT-PCR	–	–	71%	Anticoagulated whole blood	OS	48.69±10.38	8
Flavin (2009) (54)	miR-29b	Ireland	50	Stem-Loop RT-PCR	–	84%		FFPE	OS	–	7
Lee (2012) (59)	miR-30	Korea	68	qRT-PCR	median	–	–	FFPE	OS/PFS	–	8
Sestito (2015) (58)	miR-30a	Italy	39	qRT-PCR	median	–	–	tissue	PFS	–	7
Zhao (2013) (33)	miR-30a	China	30	qRT-PCR	expression level	86%	100%	FFPE	OS	mean 54 (range 29-74)	8
Wang (2013) (34)	miR-30a	China	69	qRT-PCR	ROC	100%	100%	FFPE	OS	21-74	9
Cao J (2014) (35)	miR-335	China	36	qRT-PCR	median	72%	72%	tissue	OS/PFS	mean 57	8
Welponer (2020) (46)	miR-34	Austria	228	qRT-PCR	miR-34a: 60th percentile; miR-34b: 86th percentile, miR-34c: low/ high:80th percentile	63%	63%; high grade serous 56%	tissue	OS/PFS	<61.5y 114; >61.5y 114	9
Schmid (2016) (47)	miR-34a	Austria	133	qRT-PCR	20th percentile	77%	50%	tissue	OS/PFS	<62.3y 66; >62.3y 67	9
Meng (2015) (52)	miR-429	Germany	180	qRT-PCR	median	82%	–	serum	OS	mean 60 (range 25–91)	9
Zou (2017) (36)	miR-429	China	72	qRT-PCR	–	81%	56%	tissue	OS/PFS	≤50y 38; >50y 36	8
Wang (2016) (37)	miR-433	China	115	qRT-PCR	median	57%	–	tissue	OS	≤40y 62; >40y 53	9
Zhang W (2020) (38)	miR-484	China	113	qRT-PCR	ROC	43%	42%	exosome	OS/PFS	<50y 51; ≥50y 62	8
Liu (2015) (44)	miR-506	TCGA	468	qRT-PCR	mean	–	–	tissue	OS/PFS	–	8
Zhang Y (2020) (45)	miR-506	TCGA	322	qRT-PCR	–	92%	–	tissue	PFS	58.75 ± 11.46	8
Zhang J (2016) (39)	miR-520g	China	116	qRT-PCR	–	87%	71%	tissue	OS/PFS	<50y 43; ≥50y 73	8
Wei (2018) (40)	miR-532	China	145	qRT-PCR	–	42%	–	tissue	OS	–	8
Zhou (2017) (41)	miR-595	China	166	qRT-PCR	median	39%	45%	tissue	OS	<55y 77; ≥55y 89	9
Zhang X (2016) (42)	miR-613	China	236	qRT-PCR	median	50%	64%	tissue	OS/PFS	≤55y 118; >55y 118	9

miR, microRNA; TCGA, The Cancer Genome Atlas; qRT-PCR, quantitative reverse transcription-polymerase chain reaction; ISH, immunohistochemistry; ROC, receiver operating characteristic curve; OS, overall survival; PFS, progression-free survival; FFPE, Formalin Fixed Paraffin Embedded; FIGO stage, International Federation of Gynecology and Obstetrics (FIGO) stage.

### 2.3 Classification of miRNA expression

For the primary meta-analysis, miRNAs were categorized into “up-regulated” or “down-regulated” groups based on their reported expression levels in ovarian carcinoma compared with normal or benign tissues. This classification was essential because conducting a pooled analysis of all miRNAs together would be biologically confounded, given that they include both tumor-promoting and tumor-suppressing species with opposing functions. To ensure an accurate and consistent classification, we employed a hierarchical, evidence-based approach, as detailed below, to address the fact that not all included prognostic studies provided direct comparative data.

First, for miRNAs with well-documented roles in ovarian cancer, the expression status was adopted from a comprehensive review (2), which integrates evidence from comparisons with both normal and benign tissues. Second, when a prognostic study included in our meta-analysis directly reported a comparative analysis of miRNA expression between tumor and control tissues (normal or benign), this primary data was used to supplement and corroborate the classification established in Tier 1. For the miRNAs where both sources (the review and included prognostic studies) provided expression data, the pattern documented in the review (Tier 1) was taken as the definitive reference. In this study, no instances of contradictory expression direction were observed between these two tiers of evidence. Third, for miRNAs not covered by the aforementioned sources, targeted literature searches were conducted to ascertain their consistently reported expression profile in ovarian cancer. Their classification was then based on external evidence from independent studies.

### 2.4 Statistical analyses

The main outcomes of this meta-analysis were the HRs of miRNAs associated with OS and PFS in ovarian cancer. The miRNAs were categorized as either up-regulated or down-regulated based on their expression levels in ovarian cancer samples compared to normal samples (17). Additionally, the miRNAs were grouped according to their respective families. Given that many of the identified miRNAs were reported in only one or two studies, which was deemed insufficient for robust analysis, only those miRNAs that appeared in three or more publications were included in the calculation of synthesized HR of each miRNA family (12). The pooled HR for each miRNA or miRNA family was calculated by combining the log-transformed HRs and their standard errors from the included studies using the inverse variance method, which assigns greater weight to studies with more precise estimates such as those with smaller standard errors. The degree of variability due to differences between the studies included in the analysis was assessed using the *I²* statistic. A fixed-effects model was applied when low heterogeneity was observed (*I²* < 50%), while a random-effects model was used to estimate the pooled hazard ratios (HRs) and their corresponding confidence intervals (CIs) when higher levels of heterogeneity were detected. In cases of significant heterogeneity, subgroup analyses were performed to explore potential sources of variation, considering factors such as study region, sample source, International Federation of Gynecology and Obstetrics (FIGO) stage and histotype. Sensitivity analyses were also conducted to evaluate the impact of individual studies on the overall results by systematically excluding one study at a time. To assess publication bias, the Egger’s tests (18) were performed, alongside a visual inspection of funnel plots. Trim and-fill analyses were further conducted to control for potential publication biases when P-values for Egger’s tests were less than 0.05. All meta-analyses were conducted using STATA software (version 14.0) and R software (version 4.3.0). Statistical significance was set at a two-sided P value threshold of 0.05 for all analyses.

## 3 Results

### 3.1 Literature search and basic information

**Figure 1:**
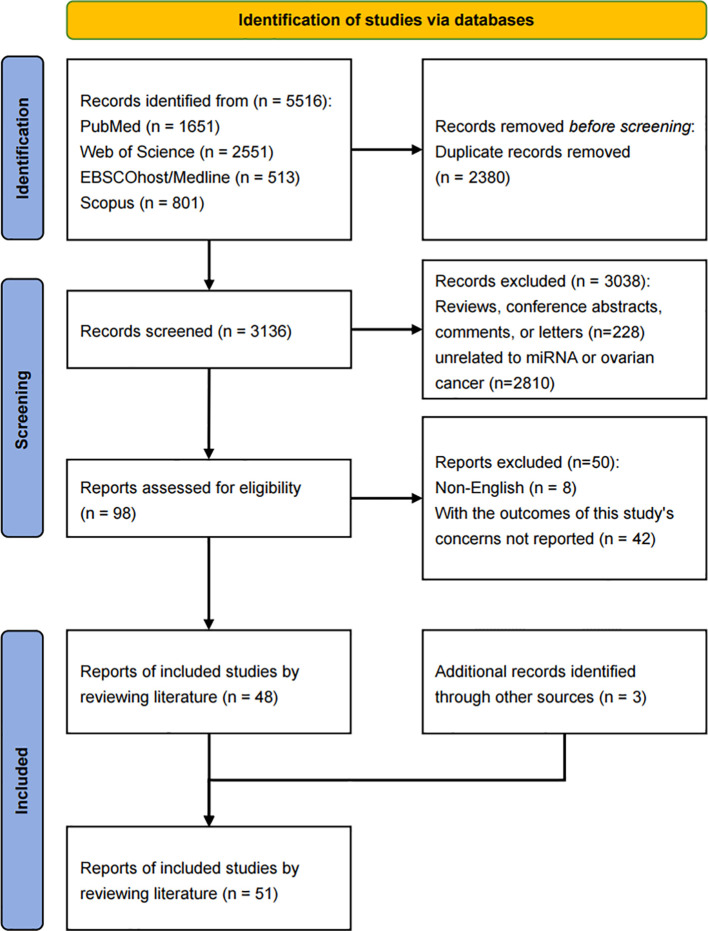
PRISMA flowchart.

A total of 5,516 titles and abstracts were initially retrieved from the four databases. After removing 2,380 duplicate records, 3,136 records remained for further screening. Of these, 3,038 records were excluded for the following reasons: 228 were reviews, conference abstracts, comments, or letters, and 2,810 were unrelated to miRNA or ovarian cancer. After screening 98 records and conducting a full-text review, 48 studies were deemed eligible for inclusion in this systematic review and meta-analysis. Additionally, three studies were identified through reference list checks of relevant articles. As a result, 51 records were ultimately included in the meta-analysis. (**Figure 1**). The agreement rate between the authors for study selection and data extraction was high and moderate (κ=0.81 and 0.68).

In total, 6,916 ovarian cancer patients and 181 types of miRNAs were included in the final analysis. All included studies were longitudinal cohort studies. Among the 51 included studies, 26 originated from China (7, 19–43), 3 originated from public databases (44–46), 22 originated from other countries, including Austria (47, 48), Denmark (49), France (50), Germany (8, 51–53), India (54), Ireland (55), Italy (4, 10, 56–59), Korea (9, 60), Norway (61), Spain (62), Turkey (63) and the United States (64). Quantitative real time-polymerase chain reaction (qRT-PCR) was performed to detect miRNA expression in the majority of the included studies (48/52). Thirty-two studies detected the miRNA expression in tissue sample, 10 studies detected in formalin-fixed paraffin embedded (FFPE) sample, and the remaining 9 studies utilized blood-based samples. In terms of the type of miRNA, 12 articles reported miRNA-200 family, followed by 6 articles on miRNA-30 family and 4 articles on let-7 family. Four studies focused on ovarian cancer at FIGO stage III-IV, while five studies specifically examined the serous histotype. In terms of quality assessment, 29 articles scored 9 points, 19 articles scored 8 points, and 3 articles scored 7 points (**Table 1**). Thus, the quality of articles included in this meta-analysis was relatively high with a mean score 8.5.

### 3.2 miRNAs and prognosis of ovarian cancer

The association between miRNAs and OS in ovarian cancer has been reported in 47 studies. Among these, 27 studies focused on up-regulated miRNAs in ovarian cancer samples compared to normal samples, while 25 studies found down-regulated miRNAs. The relationship between miRNAs and PFS in ovarian cancer was reported in 26 studies. Of these, 17 studies reported up-regulated miRNAs, and 15 studies found down-regulated miRNAs. The synthesized HR for up-regulated miRNAs associated with OS and PFS in ovarian cancer was 0.92 (95% CI: 0.82-1.02) and 0.92 (95% CI: 0.78-1.06), respectively. For down-regulated miRNAs, the synthesized HR for OS and PFS was 0.59 (95% CI: 0.50-0.69) and 0.58 (95% CI: 0.43-0.74), respectively (**Supplementary Table S3**).

Subgroup analyses were conducted based on region (China vs. other countries) and sample source (tissue, blood, and formalin-fixed paraffin embedded [FFPE]). Significant regional differences were observed in the association between up-regulated miRNAs and overall survival (OS) in ovarian cancer. Specifically, elevated expression of up-regulated miRNAs was significantly associated with improved OS in Chinese ovarian cancer patients compared to those from other regions (HR 0.43 [95% CI: 0.20-0.65] vs. 1.01 [95% CI: 0.90-1.11], P < 0.01). Similarly, down-regulated miRNAs were also significantly associated with better OS in Chinese patients (HR 0.35 [95% CI: 0.27-0.42] vs. 0.71 [95% CI: 0.59-0.84], P < 0.01). Regarding sample sources, a significant difference was found in the predictive effect of down-regulated miRNAs. miRNAs extracted from tissue samples showed a stronger predictive value for ovarian cancer prognosis compared to those extracted from FFPE samples (HR 0.55 [95% CI: 0.44-0.66] vs. 0.97 [95% CI: 0.65-1.29], P = 0.046). Similar results were observed for PFS in ovarian cancer. (**Supplementary Table S3**).

The association between miRNAs and OS in FIGO stage III-IV ovarian cancer has been explored in four studies. The serous carcinoma HR for upregulated miRNAs associated with OS and PFS in FIGO stage III-IV ovarian cancer was 0.91 (95% CI: 0.65-1.16) and 1.03 (95% CI: 0.83-1.24), respectively. Regarding histotype, five studies have investigated the relationship between miRNAs and OS in serous carcinoma. The serous carcinoma HR for upregulated miRNAs associated with OS in serous carcinoma was 0.51 (95% CI: 0.11-0.92). (**Supplementary Table S4**).

The possibility of publication bias of the up-regulated miRNAs in the prognosis of ovarian cancer was low, as evidenced by the Egger’s test and the symmetrical distribution of the funnel plot. In addition, potential publication biases were found in the meta-analysis of down-regulated miRNAs in the prognosis of ovarian cancer (P = 0.003 for OS and P = 0.025 for PFS). After controlling the potential publication biases by trim-and-fill analyses the results changed significantly both in OS (HR 0.82 [95%CI: 0.65-1.05]) and PFS (HR 0.92 [95%CI: 0.60-1.40]).

### 3.3 miRNA-200 family

**Figure 2:**
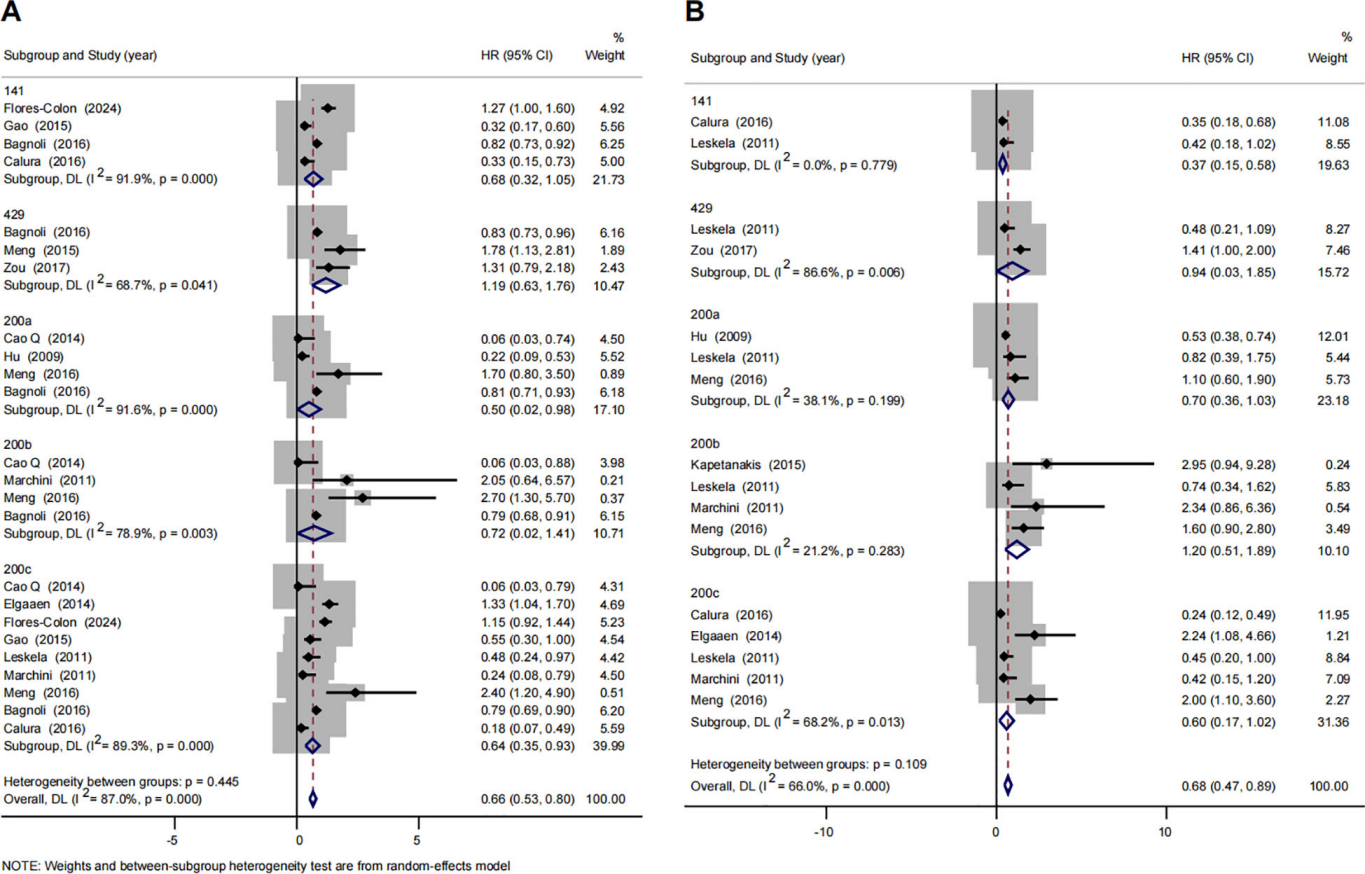
Forest plots of subgroup analysis regarding specific miR-200 family member expression and prognosis of ovarian cancer. **(A)**  overall survival, **(B)** progression-free survival. Note: The plot displays the hazard ratios (HRs) and 95% confidence intervals (CIs) for individual studies and the synthesized HR for each member of the miRNA-200 family (miR-141, -429, -200a, -200b, -200c) and the family overall. The horizontal lines through the squares represent the 95% CIs. The red dashed vertical line indicates the point estimate of the pooled hazard ratio for the overall miRNA-200 family. Diamond symbols represent the pooled HR and 95% CI for each subgroup and the overall analysis. An HR < 1 suggests that higher expression of the miRNA is associated with better overall survival, whereas an HR > 1 suggests that higher expression is associated with poorer overall survival.

Among the various miRNA families, 41 have been identified (**Supplementary Table S5**), with 8 families selected for this meta-analysis, as their miRNAs were reported in at least three studies. These families include miRNA-181, miRNA-199, miRNA-200, miRNA-29, miRNA-30, miRNA-96, miRNA-34, and let-7 (**Supplementary Table S6**). Thirteen studies examined the association between the miRNA-200 family and survival outcomes in ovarian cancer. Based on previous studies, the miRNAs included in the miRNA-200 family were miRNA-141, miRNA-429, miRNA-200a, miRNA-200b, and miRNA-200c. The association between miRNA-200 family and OS was reported in 12 studies, yielding a synthesized HR of 0.66 (95% CI: 0.53-0.80). Stratified analysis by individual miRNA members showed that elevated expression of miRNA-200a (HR 0.50 [95% CI: 0.02-0.98]) and miRNA-200c (HR 0.64 [95% CI: 0.35-0.93]) were significantly associated with improved OS. Additionally, elevated expression of the miRNA-200 family was found to be significantly associated with better PFS (HR 0.68 [95% CI: 0.47-0.89]). Further stratification revealed that increased expression of miRNA-141 (HR 0.37 [95% CI: 0.15-0.58]) was also significantly linked to improved PFS in ovarian cancer (**Figure 2**).

Subgroup analysis showed that the elevated expression of miRNA-200 family was subsequently significantly associated with better OS in Chinese ovarian patients compared to their counterparts from other regions (HR 0.32 [95%CI: 0.06-0.58] vs. 0.77 [95%CI: 0.63-0.91], P = 0.003) (**Supplementary Table S7**). In addition, When we divided miRNA-200 family into 2 subgroups based on their chromosomal location, Chr1 (miRNA-200a, miRNA-200b, and miRNA-429) and Chr12 (miRNA-141 and miRNA-200c), we found that significant statistical heterogeneity still exists (**Supplementary Figure S1**). The results of the sensitivity analysis are presented in **Supplementary Figure S2**. After excluding each study, the results did not significantly change. Furthermore, low risks of publication bias were identified in synthesized HR of miRNA-200 family both in the OS and PFS of ovarian cancer, as evidenced by the symmetrical distribution of the funnel plot (**Supplementary Figure S3**). Moreover, the results of the Egger’s tests suggested a low probability of significant publication bias (**Supplementary Table S8**).

### 3.4 miRNA-30 family

**Figure 3:**
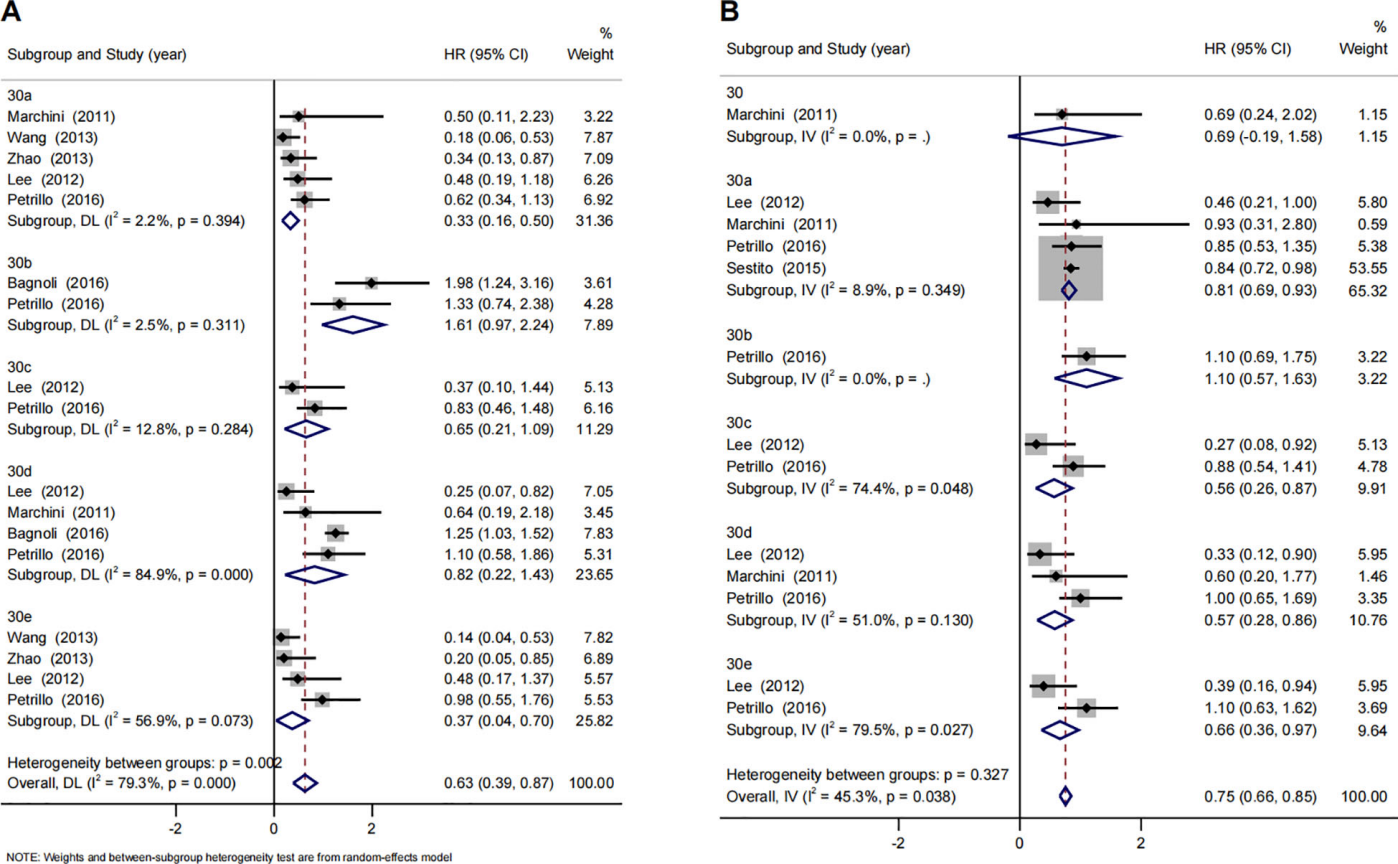
Forest plots of subgroup analysis regarding specific miR-30 family member expression and prognosis of ovarian cancer. **(A)**  overall survival, **(B)** progression-free survival. Note: The plot displays the hazard ratios (HRs) and 95% confidence intervals (CIs) for individual studies and the synthesized HR for each member of the miRNA-30 family (miR-30a, -30b, -30c, -30d, -30e) and the family overall. The horizontal lines through the squares represent the 95% CIs. The red dashed vertical line indicates the point estimate of the pooled hazard ratio for the overall miRNA-30 family. Diamond symbols represent the pooled HR and 95% CI for each subgroup and the overall analysis. An HR < 1 suggests that higher expression of the miRNA is associated with better overall survival, whereas an HR > 1 suggests that higher expression is associated with poorer overall survival.

Seven studies assessed the association between miRNA-30 family and survival outcome of ovarian cancer. The association of miRNA-30 family and OS of ovarian cancer was reported by 6 studies with the combined effect size estimate (HR 0.63 [95%CI: 0.39-0.87]) (**Supplementary Table S6**). Stratified analysis by miRNA-30 family member types revealed that elevated expression level of miRNA-30a (HR 0.33 [95%CI: 0.16-0.50]) and miRNA-30e (HR 0.37 [95%CI: 0.04-0.70]) were subsequently significantly associated with better OS of ovarian cancer. Also, We found that elevated expression level of miRNA-30 family had a significant association with better PFS (HR 0.75 [95%CI: 0.66-0.85]). Elevated expression level of miRNA-30a (HR 0.81 [95%CI: 0.69-0.93]) miRNA-30c (HR 0.56 [95%CI: 0.26-0.87]), miRNA-30d (HR 0.57 [95%CI: 0.28-0.86]) and miRNA-30e (HR 0.66 [95%CI: 0.36-0.97]) were subsequently significantly associated with better PFS of ovarian cancer after stratified analysis by miRNA-30 family (**Figure 3**). The results of the sensitivity analysis are presented in **Supplementary Figure S4**. After excluding each study, the results did not significantly change. The Egger’s test and funnel plot showed a significant publication bias among those studies reported miRNA-30 family and OS of ovarian cancer (P = 0.001) (**Supplementary Table S8**). As shown in **Supplementary Figure S5**, after controlling the potential publication biases by trim-and-fill analyses (8 studies added), the synthesized HR changed significantly (HR 1.09 [95%CI: 0.69-1.73]).

### 3.5 Other miRNA family

Four studies evaluated the association between the let-7 family and ovarian cancer prognosis. The results showed that the association between the let-7 family and overall survival (OS) and progression-free survival (PFS) in ovarian cancer was not statistically significant, with hazard ratios (HR) of 0.83 (95% CI: 0.53, 1.14) for OS and 0.83 (95% CI: 0.53, 1.13) for PFS. Similarly, the combined association between miRNA-181, miRNA-199, miRNA-29, miRNA-34 and miRNA-96 family and the prognosis of ovarian cancer is also not statistically significant (**Supplementary Table S6**).

## 4 Discussion

Studies reported the association between miRNA and prognosis of ovarian cancer have shown varied results, likely due to multiple influencing factors. Investigating these factors in detail can provide a more comprehensive understanding of the prognostic value of miRNA. However, individual studies may lack the necessary sample size for a thorough investigation across different miRNA family, regions, and sample sources. Previous meta-analyses exploring the association between miRNAs and ovarian cancer prognosis have been either based on a single database or focused on specific miRNAs. Therefore, this review is the most comprehensive systematic review and meta-analysis addressing the association between miRNAs and the prognosis of ovarian cancer.

The mechanisms by which miRNAs influence the prognosis of ovarian cancer are complex and multifaceted. Given that our meta-analysis identified the miR-200 and miR-30 families as having significant prognostic value, we focus on their established roles in ovarian cancer pathobiology. The miR-200 family, often reported as tumor suppressors, is critically involved in inhibiting epithelial-mesenchymal transition (EMT) and metastasis by targeting key transcriptional repressors of E-cadherin, such as ZEB1 and ZEB2 (65, 66). This action helps to maintain the epithelial phenotype and is associated with better survival outcomes, consistent with our prognostic findings. Conversely, the miR-200 family can also exhibit oncogenic functions in certain contexts, for instance, by promoting proliferation and chemoresistance, which may explain the heterogeneous associations observed across different studies and patient populations. Regarding the miR-30 family, its members frequently act as tumor suppressors. For example, miR-30d has been shown to suppress TGF-β1-induced EMT by directly targeting the transcription factor Snail, thereby inhibiting cell invasion and migration (67). Similarly, other members like miR-30a can promote apoptosis and sensitize ovarian cancer cells to chemotherapeutic agents by targeting genes involved in survival pathways (68). Overall, the prognostic significance of specific miRNAs, including the miR-200 and miR-30 families, likely stems from their integrated regulation of complex networks governing key oncogenic processes such as cell proliferation, EMT, metastasis, and chemoresistance. Given that individual miRNAs can regulate hundreds of target genes and are embedded in complex regulatory networks, firm conclusions about the precise molecular mechanisms underlying their prognostic value remain challenging and are an area for future functional validation.

This study found no significant association between up-regulated miRNAs and ovarian cancer prognosis in the overall meta-analysis. It is important to clarify that this specific analysis only included studies that categorized miRNA expression into “high” versus “low” groups, allowing for the calculation of a pooled HR for this defined comparison. Studies that reported miRNA expression solely as a continuous variable were excluded from this particular synthesis, as they could not be categorized into an “up-regulated” group. The overall result (HR 0.92, 95% CI: 0.82-1.02) suggests that when diverse miRNAs are combined based solely on their “high” expression status, their collective prognostic effect is not significant, which highlights the importance of our subsequent analyses focusing on specific miRNA families and subgroups. However, subgroup analysis revealed that up-regulated miRNAs were significantly associated with prognosis in China, suggesting their potential prognostic value specifically in this region. The observed regional differences in miRNA prognostic performance between Chinese and other populations could be attributed to several factors. First, genetic variations across ethnic groups may influence both miRNA expression patterns and their interactions with target genes. Second, differences in tumor biology and histotype distribution among populations could contribute to these disparities. Additionally, variations in treatment protocols, environmental exposures, and lifestyle factors across regions might modify the prognostic significance of specific miRNAs. However, it is important to note several limitations in interpreting these regional differences. The individual patient data, including detailed clinicopathological characteristics, treatment histories, and genetic backgrounds, were not available from the original studies, which prevented us from conducting more refined analyses to identify the exact sources of these regional disparities. Therefore, our findings should be interpreted as identifying an interesting phenomenon that requires validation through future studies specifically designed to explore ethnic and regional variations in miRNA prognostic value. The initial finding of a significant association between down-regulated miRNAs and better prognosis could have been influenced by publication bias. After controlling for this bias using the trim-and-fill method, the hazard ratio changed notably, indicating that the true effect might be weaker than initially estimated. These results suggest that the true nature of this association may require further validation through the inclusion of additional negative findings. The pooled analysis results indicate that the higher expression of miRNAs significantly improved the OS in women with serous carcinoma. This suggests that miRNAs may have potential value in the prognosis of serous carcinoma. However, data for other subtypes, such as mucinous and clear cell carcinoma, could not be extracted from the original studies for analysis. Similarly, only HR data for FIGO stage III-IV ovarian cancer could be obtained from the original studies, and the number of available studies is relatively limited. Therefore, this result still needs further validation. This is also one of the limitation of this study.

In this study, miRNA-200 family might have potential prognostic value of ovarian cancer, which is consistent with the previous studies (13, 14). This result supports the growing body of evidence highlighting the potential role of the miRNA-200 family as a prognostic biomarker in ovarian cancer. Subgroup analyses were carried out since the high heterogeneity between studies. While differences in the prognostic effects of individual miRNAs within the miRNA-200 family (such as miRNA-200a, miRNA-200c, and miRNA-141) were observed, the elevated expression of these miRNAs was generally associated with better OS and PFS, suggesting that the miRNA-200 family could serve as an important biomarker for ovarian cancer prognosis. In addition, although subgroup and sensitivity analyses confirmed the stability of the results, the significant statistical heterogeneity between miRNA-200 family members was not fully resolved. This heterogeneity persisted even after subgroup analysis by region, sample size, sample source and different chromosomal location. However, the *I²* decreased after analyzing by region and chromosomal location, suggesting that these factors may contribute to the variability. Beyond these methodological and demographic factors, the inherent biological diversity within the miR-200 family is a likely source of heterogeneity. Although classified as a family, individual members (e.g., miR-141, -200a, -200b, -200c, -429) can regulate distinct sets of target genes and exert divergent, effects on tumor progression and patient prognosis, which would not be fully accounted for by pooling them together in a meta-analysis. Studies have reported that the expression levels of the miRNA-200 family vary by tumor stage and histologic grade (7, 26). However, these factors could not be extracted from individual studies, preventing the identification of all sources of heterogeneity.

The pooled analysis of studies demonstrated that higher expression of the miRNA-30 family significantly improved the OS and PFS in women with ovarian cancer. The subgroup analyses of miRNA-30 family members revealed that the improved OS existed for enhanced expression of miRNA-30a and miRNA-30e. The improved PFS existed for enhanced expression of all miRNA-30a, miRNA-30c, miRNA-30d and miRNA-30e. Studies suggest that the miR-30 family, through targets like ATF3, MYC, bHLH transcription factors, and TET3, plays a key role in regulating ovarian cancer progression, with miR-30d showing potential as a prognostic biomarker by inhibiting TGF-β1-induced EMT (69). However, the finding of publication bias, as evidenced by a statistically significant result in Egger’s test (P = 0.001) supplemented by visual inspection of the funnel plot, suggests that the prognostic effect of the miR-30 family on OS in ovarian cancer may be overestimated. In contrast, the risk of publication bias for the prognostic effect on PFS is relatively low, and its heterogeneity is also smaller. This indicates that the miR-30 family may have a more stable and reliable role in predicting PFS in ovarian cancer.

The strengths of our study are as follows. First, this is the most comprehensive study to assess the potential prognostic value of miRNA in ovarian cancer. Second, our study included studies from various countries and regions. The integration of global data may provide a more comprehensive estimation of the association between overall miRNAs and each miRNA family and the prognosis of ovarian cancer. In addition to the findings, this study has several limitations. First, the overall results were not controlled for prognostic factors such as stage, grade and histology as this information could not be obtained from individual studies for the analysis of meta regression. Second, there was high heterogeneity among the studies, both overall and within subgroups, due to variations in ethnicity, age component of each cohort, tumor characteristics. These factors could not be fully extracted from each study, limiting our ability to pinpoint all sources of heterogeneity. Third, since most miRNAs have been reported in fewer than three studies, which is insufficient for inclusion in the meta-analysis of each miRNA family, these miRNAs were not included as primary results in this study. However, preliminary meta-analysis suggests that they may have potential prognostic value for ovarian cancer, such as the miRNA-34 family and the miRNA-17–92 family (reported in only two studies). These results need further validation. Fourth, only articles written in English were included.

## 5 Conclusion

In conclusion, while up-regulated miRNAs demonstrate potential prognostic value specifically in Chinese women with ovarian cancer, this association appears to be influenced by regional factors that warrant further investigation. Specifically, miRNAs may serve as valuable indicators for the prognosis of serous carcinoma. Different miRNA families, especially miR-200 and miR-30, have the potential to serve as prognostic biomarkers for ovarian cancer. The clinical application of these biomarkers could include developing standardized assays for risk stratification, potentially helping to identify patient subgroups with distinct prognostic outcomes who might benefit from different management strategies. The prognostic value of down-regulated miRNAs need further validation since the potential over-estimation due to publication bias. Further research, particularly large-scale, prospective studies designed to validate these miRNA signatures in well-defined clinical cohorts, is needed to validate these findings, especially those miRNAs that have received less attention and to firmly establish their role in clinical decision-making.

## Data Availability

The original contributions presented in the study are included in the article/**Supplementary Material**. Further inquiries can be directed to the corresponding author.

## References

[1] Siegel RL Giaquinto AN Jemal A . Cancer statistics, 2024. CA Cancer J Clin. (2024) 74:12–49. doi: 10.3322/caac.21820, PMID: 38230766

[2] Aboutalebi H Bahrami A Soleimani A Saeedi N Rahmani F Khazaei M . The diagnostic, prognostic and therapeutic potential of circulating micrornas in ovarian cancer. Int J Biochem Cell Biol. (2020) 124:105765. doi: 10.1016/j.biocel.2020.105765, PMID: 32428568

[3] Gaillard S Lacchetti C Armstrong DK Cliby WA Edelson MI Garcia AA . Neoadjuvant chemotherapy for newly diagnosed, advanced ovarian cancer: asco guideline update. J Clin Oncol. (2025) 43:868–91. doi: 10.1200/JCO-24-02589, PMID: 39841949 PMC11934100

[4] Bagnoli M Canevari S Califano D Losito S Di Maio M Raspagliesi F . Development and validation of a microrna-based signature (mirovar) to predict early relapse or progression of epithelial ovarian cancer: a cohort study. Lancet Oncol. (2016) 17:1137–46. doi: 10.1016/S1470-2045(16)30108-5, PMID: 27402147

[5] Shang R Lee S Senavirathne G Lai EC . Micrornas in action: biogenesis, function and regulation. Nat Rev Genet. (2023) 24:816–33. doi: 10.1038/s41576-023-00611-y, PMID: 37380761 PMC11087887

[6] Cortez MA Bueso-Ramos C Ferdin J Lopez-Berestein G Sood AK Calin GA . Micrornas in body fluids–the mix of hormones and biomarkers. Nat Rev Clin Oncol. (2011) 8:467–77. doi: 10.1038/nrclinonc.2011.76, PMID: 21647195 PMC3423224

[7] Cao Q Lu K Dai S Hu Y Fan W . Clinicopathological and prognostic implications of the mir-200 family in patients with epithelial ovarian cancer. Int J Clin Exp Pathol. (2014) 7:2392–401., PMID: 24966949 PMC4069884

[8] Meng X Mueller V Milde-Langosch K Trillsch F Pantel K Schwarzenbach H . Diagnostic and prognostic relevance of circulating exosomal mir-373, mir-200a, mir-200b and mir-200c in patients with epithelial ovarian cancer. ONCOTARGET. (2016) 7:16923–35. doi: 10.18632/oncotarget.7850, PMID: 26943577 PMC4941360

[9] Kim TH Song J Park H Jeong J Kwon A Heo JH . Mir-145, targeting high-mobility group a2, is a powerful predictor of patient outcome in ovarian carcinoma. Cancer Lett. (2015) 356:937–45. doi: 10.1016/j.canlet.2014.11.011, PMID: 25444913

[10] Calura E Paracchini L Fruscio R Difeo A Ravaggi A Peronne J . A prognostic regulatory pathway in stage i epithelial ovarian cancer: new hints for the poor prognosis assessment. Ann Oncol. (2016) 27:1511–9. doi: 10.1093/annonc/mdw210, PMID: 27194815

[11] Kossai M Leary A Scoazec JY Genestie C . Ovarian cancer: a heterogeneous disease. Pathobiology. (2018) 85:41–9. doi: 10.1159/000479006, PMID: 29020678

[12] Ferreira P Roela RA Lopez R Del PEM . The prognostic role of microrna in epithelial ovarian cancer: a systematic review of literature with an overall survival meta-analysis. Oncotarget. (2020) 11:1085–95. doi: 10.18632/oncotarget.27246, PMID: 32256980 PMC7105164

[13] Shi C Zhang Z . The prognostic value of the mir-200 family in ovarian cancer: a meta-analysis. Acta Obstet Gynecol Scand. (2016) 95:505–12. doi: 10.1111/aogs.12883, PMID: 26910180

[14] Shi M Mu Y Zhang H Liu M Wan J Qin X . Microrna-200 and microrna-30 family as prognostic molecular signatures in ovarian cancer: a meta-analysis. Med (Baltimore). (2018) 97:e11505. doi: 10.1097/MD.0000000000011505, PMID: 30095616 PMC6133642

[15] Page MJ Mckenzie JE Bossuyt PM Boutron I Hoffmann TC Mulrow CD . The prisma 2020 statement: an updated guideline for reporting systematic reviews. BMJ. (2021) 372:n71. doi: 10.1136/bmj.n71, PMID: 33782057 PMC8005924

[16] Wells G . The Newcastle-Ottawa Scale (NOS) for assessing the quality of nonrandomised studies in meta-analyses. In: Symposium Systematic Reviews: Beyond Basics. (2014).

[17] Huang Y Gu M Tang Y Sun Z Luo J Li Z . Systematic review and meta-analysis of prognostic microrna biomarkers for survival outcome in laryngeal squamous cell cancer. Cancer Cell Int. (2021) 21:316. doi: 10.1186/s12935-021-02021-8, PMID: 34158050 PMC8220842

[18] Egger M Davey SG Schneider M Minder C . Bias in meta-analysis detected by a simple, graphical test. BMJ. (1997) 315:629–34. doi: 10.1136/bmj.315.7109.629, PMID: 9310563 PMC2127453

[19] Wang M Zhang B Dai X Zhang Y Lian W . Decreased expression of *microrna*-*433* is associated with the prognosis of epithelial ovarian cancer. Int J Clin Exp Pathol. (2016) 9:3606–11.

[20] Gong L Wang C Gao Y Wang J . Decreased expression of microrna-148a predicts poor prognosis in ovarian cancer and associates with tumor growth and metastasis. BioMed Pharmacother. (2016) 83:58–63. doi: 10.1016/j.biopha.2016.05.049, PMID: 27470550

[21] Zhang X Zhang H . Diminished mir-613 expression as a novel prognostic biomarker for human ovarian cancer. Eur Rev Med Pharmacol Sci. (2016) 20:837–41., PMID: 27010138

[22] Guo TY Xu HY Chen WJ Wu MX Dai X . Downregulation of mir-1294 associates with prognosis and tumor progression in epithelial ovarian cancer. Eur Rev Med Pharmacol Sci. (2018) 22:7646–52. doi: 10.26355/eurrev_201811_16381, PMID: 30536306

[23] Zou J Liu L Wang Q Yin F Yang Z Zhang W . Downregulation of mir-429 contributes to the development of drug resistance in epithelial ovarian cancer by targeting zeb1. Am J Transl Res. (2017) 9:1357–68., PMID: 28386361 PMC5376026

[24] Fan Y Fan J Huang L Ye M Huang Z Wang Y . Increased expression of microrna-196a predicts poor prognosis in human ovarian carcinoma. Int J Clin Exp Pathol. (2015) 8:4132–7., PMID: 26097603 PMC4466990

[25] Zhang W Su X Li S Liu Z Wang Q Zeng H . Low serum exosomal mir-484 expression predicts unfavorable prognosis in ovarian cancer. Cancer biomark. (2020) 27:485–91. doi: 10.3233/CBM-191123, PMID: 32065786 PMC12662311

[26] Chen H Zhang L Zhang L Du J Wang H Wang B . Microrna-183 correlates cancer prognosis, regulates cancer proliferation and bufalin sensitivity in epithelial ovarian caner. Am J Transl Res. (2016) 8:1748–55., PMID: 27186298 PMC4859903

[27] Qin C Lou X Lv Q Cheng L Wu N Hu L . Microrna-184 acts as a potential diagnostic and prognostic marker in epithelial ovarian cancer and regulates cell proliferation, apoptosis and inflammation. Pharmazie. (2015) 70:668–73. doi: 10.1691/ph.2015.5577, PMID: 26601424

[28] Gao Y Wu J . Microrna-200c and microrna-141 as potential diagnostic and prognostic biomarkers for ovarian cancer. TUMOR Biol. (2015) 36:4843–50. doi: 10.1007/s13277-015-3138-3, PMID: 25636451

[29] Pan Q Xue M Yang Y Wan Y . Microrna-217 was downregulated in ovarian cancer and was associated with poor prognosis. Int J Clin Exp Pathol. (2016) 9:8555–9.

[30] Fu X Li Y Alvero A Li J Wu Q Xiao Q . Microrna-222-3p/gnai2/akt axis inhibits epithelial ovarian cancer cell growth and associates with good overall survival. ONCOTARGET. (2016) 7:80633–54. doi: 10.18632/oncotarget.13017, PMID: 27811362 PMC5348346

[31] Zhang J Liu L Sun Y Xiang J Zhou D Wang L . Microrna-520g promotes epithelial ovarian cancer progression and chemoresistance via dapk2 repression. ONCOTARGET. (2016) 7:26516–34. doi: 10.18632/oncotarget.8530, PMID: 27049921 PMC5041996

[32] Xie J Jiang Y Xu W Tao J . Mir-1231 correlates tumor prognosis and inhibits cell growth in ovarian cancer. Eur Rev Med Pharmacol Sci. (2020) 24:8308–13. doi: 10.26355/eurrev_202008_22627, PMID: 32894537

[33] Cao J Cai J Huang D Han Q Chen Y Yang Q . Mir-335 represents an independent prognostic marker in epithelial ovarian cancer. Am J Clin Pathol. (2014) 141:437–42. doi: 10.1309/AJCPLYTZGB54ISZC, PMID: 24515774

[34] Wei H Tang QL Zhang K Sun JJ Ding RF . Mir-532-5p is a prognostic marker and suppresses cells proliferation and invasion by targeting twist1 in epithelial ovarian cancer. Eur Rev Med Pharmacol Sci. (2018) 22:5842–50., PMID: 30280764 10.26355/eurrev_201809_15911

[35] Zhou QH Zha YM Jia LL Zhang Y . Mir-595 is a significant indicator of poor patient prognosis in epithelial ovarian cancer. Eur Rev Med Pharmacol Sci. (2017) 21:4278–82., PMID: 29077170

[36] Zhao H Ding Y Tie B Sun ZF Jiang JY Zhao J . Mirna expression pattern associated with prognosis in elderly patients with advanced opsc and occ. Int J Oncol. (2013) 43:839–49. doi: 10.3892/ijo.2013.1988, PMID: 23787480

[37] Zheng H Zhang L Zhao Y Yang D Song F Wen Y . Plasma mirnas as diagnostic and prognostic biomarkers for ovarian cancer. PloS One. (2013) 8:e77853. doi: 10.1371/journal.pone.0077853, PMID: 24223734 PMC3815222

[38] Peng D Luo M Qiu L He Y Wang X . Prognostic implications of microrna-100 and its functional roles in human epithelial ovarian cancer. Oncol Rep. (2012) 27:1238–44. doi: 10.3892/or.2012.1625, PMID: 22246341 PMC3583406

[39] Liu L Han Q Cai J Xiao M Huang D Cao J . The clinical validity of mir-126 as a prognostic marker in epithelial ovarian cancer. Med (Baltimore). (2023) 102:e33085. doi: 10.1097/MD.0000000000033085, PMID: 36862865 PMC9981431

[40] Wang Y Li L Qu Z Li R Bi T Jiang J . The expression of mir-30a* and mir-30e* is associated with a dualistic model for grading ovarian papillary serious carcinoma. Int J Oncol. (2014) 44:1904–14. doi: 10.3892/ijo.2014.2359, PMID: 24676806

[41] Li H Xu Y Qiu W Zhao D Zhang Y . Tissue mir-193b as a novel biomarker for patients with ovarian cancer. Med Sci Monit. (2015) 21:3929–34. doi: 10.12659/MSM.895407, PMID: 26675282 PMC4687946

[42] Yu X Zhang X Wang G Wang B Ding Y Zhao J . Mir-206 as a prognostic and sensitivity biomarker for platinum chemotherapy in epithelial ovarian cancer. Cancer Cell Int. (2020) 20:534. doi: 10.1186/s12935-020-01623-y, PMID: 33292230 PMC7641844

[43] Wan WN Zhang YQ Wang XM Liu YJ Zhang YX Que YH . Down-regulated mir-22 as predictive biomarkers for prognosis of epithelial ovarian cancer. Diagn Pathol. (2014) 9:178. doi: 10.1186/s13000-014-0178-8, PMID: 25257702 PMC4180346

[44] Liu G Yang D Rupaimoole R Pecot CV Sun Y Mangala LS . Augmentation of response to chemotherapy by microrna-506 through regulation of rad51 in serous ovarian cancers. J Natl Cancer Inst. (2015) 107:djv108. doi: 10.1093/jnci/djv108, PMID: 25995442 PMC4554255

[45] Zhang Y Ye Q He J Chen P Wan J Li J . Recurrence-associated multi-rna signature to predict disease-free survival for ovarian cancer patients. BioMed Res Int. (2020) 2020:1618527. doi: 10.1155/2020/1618527, PMID: 32149080 PMC7044477

[46] Flores-Colon M Rivera-Serrano M Reyes-Burgos VG Rolon JG Perez-Santiago J Marcos-Martinez MJ . Microrna expression profiles in human samples and cell lines revealed nine mirnas associated with cisplatin resistance in high-grade serous ovarian cancer. Int J Mol Sci. (2024) 25:3793. doi: 10.3390/ijms25073793, PMID: 38612604 PMC11011404

[47] Welponer H Tsibulak I Wieser V Degasper C Shivalingaiah G Wenzel S . The mir-34 family and its clinical significance in ovarian cancer. J Cancer. (2020) 11:1446–56. doi: 10.7150/jca.33831, PMID: 32047551 PMC6995379

[48] Schmid G Notaro S Reimer D Abdel-Azim S Duggan-Peer M Holly J . Expression and promotor hypermethylation of mir-34a in the various histological subtypes of ovarian cancer. BMC Cancer. (2016) 16:102. doi: 10.1186/s12885-016-2135-2, PMID: 26879132 PMC4754861

[49] Prahm KP Hogdall C Karlsen MA Christensen IJ Novotny GW Hogdall E . Identification and validation of potential prognostic and predictive mirnas of epithelial ovarian cancer. PloS One. (2018) 13:e207319. doi: 10.1371/journal.pone.0207319, PMID: 30475821 PMC6261038

[50] Kapetanakis NI Uzan C Jimenez-Pailhes AS Gouy S Bentivegna E Morice P . Plasma mir-200b in ovarian carcinoma patients: distinct pattern of pre/post-treatment variation compared to ca-125 and potential for prediction of progression-free survival. Oncotarget. (2015) 6:36815–24. doi: 10.18632/oncotarget.5766, PMID: 26416421 PMC4742212

[51] Panoutsopoulou K Avgeris M Magkou P Mavridis K Dreyer T Dorn J . Mir-181a overexpression predicts the poor treatment response and early-progression of serous ovarian cancer patients. Int J Cancer. (2020) 147:3560–73. doi: 10.1002/ijc.33182, PMID: 32621752

[52] Panoutsopoulou K Avgeris M Mavridis K Dreyer T Dorn J Obermayr E . Mir-203 is an independent molecular predictor of prognosis and treatment outcome in ovarian cancer: a multi-institutional study. Carcinogenesis. (2020) 41:442–51. doi: 10.1093/carcin/bgz163, PMID: 31586203

[53] Meng X Joosse SA Mueller V Trillsch F Milde-Langosch K Mahner S . Diagnostic and prognostic potential of serum mir-7, mir-16, mir-25, mir-93, mir-182, mir-376a and mir-429 in ovarian cancer patients. Br J Cancer. (2015) 113:1358–66. doi: 10.1038/bjc.2015.340, PMID: 26393886 PMC4815782

[54] Pal R Choudhury T Ghosh M Vernakar M Nath P Nasare VD . A signature of circulating mirnas predicts the prognosis and therapeutic outcome of taxane/platinum regimen in advanced ovarian carcinoma patients. Clin Transl Oncol. (2024) 26:1716–24. doi: 10.1007/s12094-024-03394-8, PMID: 38472557

[55] Flavin R Smyth P Barrett C Russell S Wen H Wei J . Mir-29b expression is associated with disease-free survival in patients with ovarian serous carcinoma. Int J Gynecol Cancer. (2009) 19:641–7. doi: 10.1111/IGC.0b013e3181a48cf9, PMID: 19509563

[56] Lu L Schwartz P Scarampi L Rutherford T Canuto EM Yu H . Microrna let-7a: a potential marker for selection of paclitaxel in ovarian cancer management. Gynecol Oncol. (2011) 122:366–71. doi: 10.1016/j.ygyno.2011.04.033, PMID: 21571355

[57] Petrillo M Zannoni GF Beltrame L Martinelli E Difeo A Paracchini L . Identification of high-grade serous ovarian cancer mirna species associated with survival and drug response in patients receiving neoadjuvant chemotherapy: a retrospective longitudinal analysis using matched tumor biopsies. Ann Oncol. (2016) 27:625–34. doi: 10.1093/annonc/mdw007, PMID: 26782955

[58] Marchini S Cavalieri D Fruscio R Calura E Garavaglia D Nerini IF . Association between mir-200c and the survival of patients with stage i epithelial ovarian cancer: a retrospective study of two independent tumour tissue collections. Lancet Oncol. (2011) 12:273–85. doi: 10.1016/S1470-2045(11)70012-2, PMID: 21345725

[59] Sestito R Cianfrocca R Rosano L Tocci P Semprucci E Di Castro V . Mir-30a inhibits endothelin a receptor and chemoresistance in ovarian carcinoma. Oncotarget. (2016) 7:4009–23. doi: 10.18632/oncotarget.6546, PMID: 26675258 PMC4826186

[60] Lee H Park CS Deftereos G Morihara J Stern JE Hawes SE . Microrna expression in ovarian carcinoma and its correlation with clinicopathological features. World J Surg Oncol. (2012) 10:174. doi: 10.1186/1477-7819-10-174, PMID: 22925189 PMC3449188

[61] Vilming EB Olstad OK Haug KB Brusletto B Sandvik L Staff AC . Global mirna expression analysis of serous and clear cell ovarian carcinomas identifies differentially expressed mirnas including mir-200c-3p as a prognostic marker. BMC Cancer. (2014) 14:80. doi: 10.1186/1471-2407-14-80, PMID: 24512620 PMC3928323

[62] Leskela S Leandro-Garcia LJ Mendiola M Barriuso J Inglada-Perez L Munoz I . The mir-200 family controls beta-tubulin iii expression and is associated with paclitaxel-based treatment response and progression-free survival in ovarian cancer patients. Endocr Relat Cancer. (2011) 18:85–95. doi: 10.1677/ERC-10-0148, PMID: 21051560

[63] Minareci Y Ak N Sozen H Tosun OA Kucukgergin C Aydin F . The evaluation of mir-1181 and mir-4314 as serum microrna biomarkers for epithelial ovarian cancer diagnosis and prognosis. Mol Biol Rep. (2024) 51:515. doi: 10.1007/s11033-024-09464-y, PMID: 38622482

[64] Hu X Macdonald DM Huettner PC Feng Z El NI Schwarz JK . A mir-200 microrna cluster as prognostic marker in advanced ovarian cancer. Gynecol Oncol. (2009) 114:457–64. doi: 10.1016/j.ygyno.2009.05.022, PMID: 19501389

[65] Perdigao-Henriques R Petrocca F Altschuler G Thomas MP Le MT Tan SM . Mir-200 promotes the mesenchymal to epithelial transition by suppressing multiple members of the zeb2 and snail1 transcriptional repressor complexes. Oncogene. (2016) 35:158–72. doi: 10.1038/onc.2015.69, PMID: 25798844

[66] Zaman MS Maher DM Khan S Jaggi M Chauhan SC . Current status and implications of micrornas in ovarian cancer diagnosis and therapy. J Ovarian Res. (2012) 5:44. doi: 10.1186/1757-2215-5-44, PMID: 23237306 PMC3539914

[67] Ye Z Zhao L Li J Chen W Li X . Mir-30d blocked transforming growth factor beta1-induced epithelial-mesenchymal transition by targeting snail in ovarian cancer cells. Int J Gynecol Cancer. (2015) 25:1574–81. doi: 10.1097/IGC.0000000000000546, PMID: 26501435

[68] Wang X Zhao H Wang P Zhang J Li N Liu Y . Mir-30a-5p/chd1 axis enhances cisplatin sensitivity of ovarian cancer cells via inactivating the wnt/beta-catenin pathway. Anticancer Drugs. (2022) 33:989–98. doi: 10.1097/CAD.0000000000001397, PMID: 36206129

[69] Ye Z Li J Han X Hou H Chen H Zheng X . Tet3 inhibits tgf-beta1-induced epithelial-mesenchymal transition by demethylating mir-30d precursor gene in ovarian cancer cells. J Exp Clin Cancer Res. (2016) 35:72. doi: 10.1186/s13046-016-0350-y, PMID: 27141829 PMC4855705

