# Supplementary material for: Prognostic value of micro-RNA in ovarian cancer: a systematic review and meta-analysis
Source: Front Oncol. 2026 Jan 12;15:1641602. doi: 10.3389/fonc.2025.1641602 (PMC12832368; doi:10.3389/fonc.2025.1641602)
Supplement: Supplementary file 1 [file DataSheet1.docx]

**Supplementary materials**

Supplementary materials include 7 tables and 5 figures.

**Table S1**. Checklist of the PRISMA extension for meta-analysis.

**Table S2**. Searching strategies in detail for four databases.

**Table S3.** The associations between miRNAs and survival of ovarian cancer.

**Table S4.** The associations between miRNAs and survival of different subtype of ovarian cancer.

**Table S5.** MicroRNAs included in this meta-analysis.

**Table S6.** Association between each miRNA family and the prognosis of ovarian cancer.

**Table S7.** Subgroup analysis of microRNA-200 family and microRNA-30 family.

**Table S8.** Egger’s test value for each microRNA.

**Table S1. Checklist of the PRISMA extension for meta-analysis**

| **Section and Topic** | **Item #** | **Checklist item** | **Location where item is reported** |
| --- | --- | --- | --- |
| **TITLE** | | |  |
| Title | 1 | Identify the report as a systematic review. | 1 |
| **ABSTRACT** | | |  |
| Abstract | 2 | See the PRISMA 2020 for Abstracts checklist. | 1 |
| **INTRODUCTION** | | |  |
| Rationale | 3 | Describe the rationale for the review in the context of existing knowledge. | 2 |
| Objectives | 4 | Provide an explicit statement of the objective(s) or question(s) the review addresses. | 2 |
| **METHODS** | | |  |
| Eligibility criteria | 5 | Specify the inclusion and exclusion criteria for the review and how studies were grouped for the syntheses. | 2 |
| Information sources | 6 | Specify all databases, registers, websites, organisations, reference lists and other sources searched or consulted to identify studies. Specify the date when each source was last searched or consulted. | 2 |
| Search strategy | 7 | Present the full search strategies for all databases, registers and websites, including any filters and limits used. | 2 |
| Selection process | 8 | Specify the methods used to decide whether a study met the inclusion criteria of the review, including how many reviewers screened each record and each report retrieved, whether they worked independently, and if applicable, details of automation tools used in the process. | 2 |
| Data collection process | 9 | Specify the methods used to collect data from reports, including how many reviewers collected data from each report, whether they worked independently, any processes for obtaining or confirming data from study investigators, and if applicable, details of automation tools used in the process. | 2 |
| Data items | 10a | List and define all outcomes for which data were sought. Specify whether all results that were compatible with each outcome domain in each study were sought (e.g. for all measures, time points, analyses), and if not, the methods used to decide which results to collect. | 2 |
|  | 10b | List and define all other variables for which data were sought (e.g. participant and intervention characteristics, funding sources). Describe any assumptions made about any missing or unclear information. | 2 |
| Study risk of bias assessment | 11 | Specify the methods used to assess risk of bias in the included studies, including details of the tool(s) used, how many reviewers assessed each study and whether they worked independently, and if applicable, details of automation tools used in the process. | 2-3 |
| Effect measures | 12 | Specify for each outcome the effect measure(s) (e.g. risk ratio, mean difference) used in the synthesis or presentation of results. | 3 |
| Synthesis methods | 13a | Describe the processes used to decide which studies were eligible for each synthesis (e.g. tabulating the study intervention characteristics and comparing against the planned groups for each synthesis (item #5)). | 3 |
|  | 13b | Describe any methods required to prepare the data for presentation or synthesis, such as handling of missing summary statistics, or data conversions. | 3 |
|  | 13c | Describe any methods used to tabulate or visually display results of individual studies and syntheses. | 3 |
|  | 13d | Describe any methods used to synthesize results and provide a rationale for the choice(s). If meta-analysis was performed, describe the model(s), method(s) to identify the presence and extent of statistical heterogeneity, and software package(s) used. | 3 |
|  | 13e | Describe any methods used to explore possible causes of heterogeneity among study results (e.g. subgroup analysis, meta-regression). | 3 |
|  | 13f | Describe any sensitivity analyses conducted to assess robustness of the synthesized results. | 3 |
| Reporting bias assessment | 14 | Describe any methods used to assess risk of bias due to missing results in a synthesis (arising from reporting biases). | 3 |
| Certainty assessment | 15 | Describe any methods used to assess certainty (or confidence) in the body of evidence for an outcome. | 3 |
| **RESULTS** | | |  |
| Study selection | 16a | Describe the results of the search and selection process, from the number of records identified in the search to the number of studies included in the review, ideally using a flow diagram. | 3 |
|  | 16b | Cite studies that might appear to meet the inclusion criteria, but which were excluded, and explain why they were excluded. | 6 |
| Study characteristics | 17 | Cite each included study and present its characteristics. | 3-6 |
| Risk of bias in studies | 18 | Present assessments of risk of bias for each included study. | 3-6 |
| Results of individual studies | 19 | For all outcomes, present, for each study: (a) summary statistics for each group (where appropriate) and (b) an effect estimate and its precision (e.g. confidence/credible interval), ideally using structured tables or plots. | 3-6 |
| Results of syntheses | 20a | For each synthesis, briefly summarise the characteristics and risk of bias among contributing studies. | 3-6 |
|  | 20b | Present results of all statistical syntheses conducted. If meta-analysis was done, present for each the summary estimate and its precision (e.g. confidence/credible interval) and measures of statistical heterogeneity. If comparing groups, describe the direction of the effect. | 3-6 |
|  | 20c | Present results of all investigations of possible causes of heterogeneity among study results. | 7-9 |
|  | 20d | Present results of all sensitivity analyses conducted to assess the robustness of the synthesized results. | 7-9 |
| Reporting biases | 21 | Present assessments of risk of bias due to missing results (arising from reporting biases) for each synthesis assessed. | 7-9 |
| Certainty of evidence | 22 | Present assessments of certainty (or confidence) in the body of evidence for each outcome assessed. | 7-10 |
| **DISCUSSION** | | |  |
| Discussion | 23a | Provide a general interpretation of the results in the context of other evidence. | 9-10 |
|  | 23b | Discuss any limitations of the evidence included in the review. | 11 |
|  | 23c | Discuss any limitations of the review processes used. | 11 |
|  | 23d | Discuss implications of the results for practice, policy, and future research. | 11 |
| **OTHER INFORMATION** | | |  |
| Registration and protocol | 24a | Provide registration information for the review, including register name and registration number, or state that the review was not registered. | 2 |
|  | 24b | Indicate where the review protocol can be accessed, or state that a protocol was not prepared. | 2 |
|  | 24c | Describe and explain any amendments to information provided at registration or in the protocol. | 2 |
| Support | 25 | Describe sources of financial or non-financial support for the review, and the role of the funders or sponsors in the review. | 12 |
| Competing interests | 26 | Declare any competing interests of review authors. | 12 |
| Availability of data, code and other materials | 27 | Report which of the following are publicly available and where they can be found: template data collection forms; data extracted from included studies; data used for all analyses; analytic code; any other materials used in the review. | 12 |

**Table S2. Searching strategies in detail for four databases**

**PubMed**

| #1 | Ovarian cancer OR Ovarian carcinoma[MeSH Major Topic] |
| --- | --- |
| *#2* | microRNA OR miRNA OR miR[MeSH Major Topic] |
| #3 | prognosis OR survive OR survival[MeSH Major Topic] |

**ISI Web of Science**

| #1 | Topic: (Ovarian cancer) OR Topic: (Ovarian carcinoma) |
| --- | --- |
| *#2* | Topic: (microRNA) OR Topic: (miRNA) OR Topic: (miR) |
| #3 | Topic: (prognosis) OR Topic: (survive) OR Topic: (survival) |
| #4 | #3 AND #2 AND #1 |

**MEDLINE (via EBSCOhost)**

| #1 | (MeSH "Ovarian Neoplasms+") OR (MeSH "Carcinoma, Ovarian Epithelial") |
| --- | --- |
| *#2* | (MeSH "MicroRNAs+") OR (MeSH "Circulating MicroRNA") |
| #3 | (MeSH "Prognosis+") OR (MeSH "Disease-Free Survival") OR (MeSH "Disease Progression+") |

**Scopus**

| #1 | Title/Abstract/Keyword (ovarian cancer OR ovarian carcinoma) |
| --- | --- |
| *#2* | Title/Abstract/Keyword (microrna OR mirna OR mir) |
| #3 | Title/Abstract/Keyword (prognosis OR survive OR survival) |

**Table S3. The associations between miRNAs and survival of ovarian cancer.**

| Category | Number of studies | HR (95%CI) | *I^2^* | P value |
| --- | --- | --- | --- | --- |
| **Up-regulated miRNAs** |  |  |  |  |
| **Overall survival** | 28 | 0.92 (0.82, 1.02) | 86% | <0.001 |
| Country |  |  |  | <0.001 |
| China | 11 | 0.43 (0.20, 0.65) | 79% |  |
| Other region | 17 | 1.01 (0.90, 1.11) | 84% |  |
| Sample source |  |  |  | 0.377 |
| tissue | 14 | 0.96 (0.84, 1.08) | 89% |  |
| blood | 4 | 1.02 (0.57, 1.47) | 63% |  |
| FFPE | 9 | 0.80 (0.59, 1.00) | 81% |  |
| **Progression-free survival** | 17 | 0.92 (0.78. 1.06) | 77% | <0.001 |
| Country |  |  |  | 0.019 |
| China | 4 | 1.50 (1.01, 1.98) | 0% |  |
| Other region | 14 | 0.90 (0.75, 1.04) | 78% |  |
| Sample source |  |  |  | 0.013 |
| tissue | 11 | 0.81 (0.58, 1.04) | 80% |  |
| blood | 2 | 2.57 (1.43, 3.72) | 0% |  |
| FFPE | 4 | 0.88 (0.71, 1.05) | 68% |  |
| **Down-regulated miRNAs** |  |  |  |  |
| **Overall survival** | 29 | 0.59 (0.50, 0.69) | 92% | <0.001 |
| Country |  |  |  | <0.001 |
| China | 16 | 0.35 (0.27, 0.42) | 48% |  |
| Other region | 9 | 0.71 (0.59, 0.84) | 93% |  |
| Sample source |  |  |  | 0.046 |
| tissue | 18 | 0.55 (0.44, 0.66) | 93% |  |
| blood | 2 | 0.69 (0.39, 0.99) | 37% |  |
| FFPE | 4 | 0.97 (0.65, 1.29) | 70% |  |
| **Progression-free survival** | 15 | 0.58 (0.43, 0.74) | 96% | 0.016 |
| Country |  |  |  | 0.006 |
| China | 4 | 0.34 (0.22, 0.45) | 27% |  |
| Other region | 10 | 0.63 (0.46, 0.80) | 96% |  |
| Sample source |  |  |  | 0.002 |
| tissue | 9 | 0.45 (0.26, 0.64) | 97% |  |
| FFPE | 3 | 0.97 (0.73, 1.21) | 50% |  |

Note: HR, hazard ratio

**Table S4. The associations between miRNAs and survival of different subtype of ovarian cancer.**

| Category | Number of studies | HR (95%CI) | *I^2^* |
| --- | --- | --- | --- |
| **FIGO stage III-IV** |  |  |  |
| Overall survival | 4 | 0.91 (0.65, 1.16) | 76% |
| Progression-free survival | 3 | 1.03 (0.83, 1.24) | 62% |
| **Serous carcinoma** |  |  |  |
| Overall survival | 5 | 0.51 (0.11, 0.92) | 91% |

Note: HR, hazard ratio; FIGO, International Federation of Gynecology and Obstetrics

**Table S5. MicroRNAs included in this meta-analysis**

| Study | MicroRNA | Sample size | Expression level compared with normal tissue | MicroRNA family | HR (95%CI) | |
| --- | --- | --- | --- | --- | --- | --- |
|  |  |  |  |  | OS | PFS |
| Marchini (2011) | 20 | 89 | Down regulated | miR-17-92 family | 0.37 (0.12, 1.17) | 0.39 (0.14, 1.08) |
| Wan (2014) | 22 | 109 | Down regulated | miR-22 family | 0.39 (0.10, 0.51) | 0.43 (0.09, 0.49) |
| Marchini (2011) | 30 | 89 | Up-regulated | miR-30 family | - | 0.69 (0.24, 2.02) |
| Flores-Colon (2024) | 96 | 486 | Up-regulated | miR-96 family | 1.51 (1.15, 1.99) | - |
| Peng (2012) | 100 | 98 | Down regulated | miR-99 family | 0.47 (0.29, 0.53) | - |
| Liu (2023) | 126 | 69 | Up-regulated | miR-126 family | 2.42 (0.94, 6.24) | - |
| Liu (2023) | 126 | 69 | Up-regulated | miR-126 family | - | 2.38 (1.03, 5.55) |
| Flores-Colon (2024) | 141 | 486 | Up-regulated | miR-200 family | 1.27 (1.00, 1.60) | - |
| Gao (2015) | 141 | 93 | Up-regulated | miR-200 family | 0.32 (0.17, 0.6) | - |
| Leskela (2011) | 141 | 72 | Up-regulated | miR-200 family | - | 0.42 (0.18, 1.02) |
| Marchini (2011) | 143 | 89 | Up-regulated | miR-143/145 family | 2.81 (0.63, 12.49) | 2.23 (0.73, 6.86) |
| Kim (2015) | 145 | 74 | Up-regulated | miR-143/145 family | 0.12 (0.01, 0.90) | - |
| Marchini (2011) | 145 | 89 | Up-regulated | miR-143/145 family | 0.14 (0.03, 0.75) | 2.24 (0.47, 10.59) |
| Gong (2016) | 148 | 102 | Down regulated | miR-148/miR-152 family | 0.59 (0.41, 0.85) | - |
| Flores-Colon (2024) | 182 | 486 | Up-regulated | miR-96 family | 1.14 (0.89, 1.45) | - |
| Pal (2024) | 182 | 208 | Up-regulated | miR-96 family | 1.25 (0.45, 3.45) | - |
| Chen (2016) | 183 | 75 | Up-regulated | miR-96 family | 3.86 (2.77, 7.96) | - |
| Flores-Colon (2024) | 183 | 486 | Up-regulated | miR-96 family | 0.84 (0.65, 1.08) | - |
| Qin (2015) | 184 | 80 | Down regulated |  | 0.22 (0.11, 0.43) | - |
| Fan (2015) | 196 | 156 | Up-regulated | miR-196 family | 2.73 (0.80, 9.64) | 2.43 (0.64, 8.54) |
| Hu (2009) | 200 | 55 | Up-regulated | miR-200 family | - | 0.53 (0.38, 0.74) |
| Panoutsopoulou (2020) | 203 | 103 | Up-regulated | miR-203 family | 2.81 (1.59, 4.97) | 2.33 (1.34, 4.04) |
| Yu (2020) | 206 | 316 | Up-regulated | miR-1/206 family | 1.27 (0.98, 1.63) | - |
| Marchini (2011) | 214 | 89 | Up-regulated | miR-214 family | 2.03 (0.35, 11.68) | 1.63 (0.45, 5.92) |
| Pal (2024) | 214 | 208 | Up-regulated | miR-214 family | 1.62 (0.01, 3.25) | - |
| Pan (2016) | 217 | 129 | Down regulated | miR-217 family | 0.39 (0.13, 0.80) | - |
| Fu (2016) | 222 | 74 | Down regulated | miR-221/222 family | 0.35 (0.16, 0.73) | - |
| Flores-Colon (2024) | 296 | 486 | Up-regulated | miR-296 family | 1.12 (0.89, 1.40) | - |
| Cao J (2014) | 335 | 36 | Down regulated | miR-335 family | 0.15 (0.05, 0.48) | 0.24 (0.09, 0.64) |
| Lee (2012) | 368 | 68 | Up-regulated | miR-367/368 family | 1.79 (0.75, 4.25) | 0.92 (0.43, 1.97) |
| Lee (2012) | 370 | 68 | Down regulated | miR-370/373 family | 1.83 (0.78, 4.31) | 1.77 (0.93, 3.34) |
| Meng (2016) | 373 | 163 | Up-regulated | miR-373/520 family | 2.10 (1.00, 4.30) | 1.60 (0.90, 2.90) |
| Bagnoli (2016) | 429 | 179 | Up-regulated | miR-200 family | 0.84 (0.73, 0.96) | - |
| Leskela (2011) | 429 | 72 | Up-regulated | miR-200 family | - | 0.48 (0.21, 1.09) |
| Meng (2015) | 429 | 180 | Up-regulated | miR-200 family | 1.78 (1.13, 2.81) | - |
| Zou (2017) | 429 | 72 | Up-regulated | miR-200 family | 1.31 (0.79, 2.18) | 1.41 (1.00, 2.00) |
| Wang (2016) | 433 | 115 | Down regulated | miR-433/miR-127 family | 0.34 (0.14, 0.79) | - |
| Bagnoli (2016) | 484 | 179 | Down regulated | miR-484 family | 1.60 (1.16, 2.21) | - |
| Zhang W (2020) | 484 | 113 | Down regulated | miR-484 family | 0.23 (0.16, 0.36) | 0.25 (0.17, 0.44) |
| Zhao (2013) | 505 | 30 | Down regulated | - | 0.12 (0.04, 0.86) | - |
| Liu (2015) | 506 | 468 | Down regulated | miR-506 family | 0.52 (0.34, 0.91) | 0.62 (0.40, 0.94) |
| Zhang Y (2020) | 506 | 322 | Down regulated | miR-506 family | - | 0.94 (0.88, 0.99) |
| Bagnoli (2016) | 507 | 179 | Down regulated | - | 0.59 (0.43, 0.81) | - |
| Zhang Y (2020) | 508 | 322 | Up-regulated | - | - | 1.32 (1.02, 1.75) |
| Zhang J (2016) | 520 | 116 | Up-regulated | miR-373/520 family | 6.16 (3.15, 11.01) | 4.05 (1.43, 11.14) |
| Pal (2024) | 591 | 208 | Up-regulated | - | 1.60 (0.74, 3.47) | - |
| Bagnoli (2016) | 592 | 179 | Down regulated | - | 0.26 (0.10, 0.66) | - |
| Zhou (2017) | 595 | 166 | Down regulated | - | 0.24 (0.14, 0.53) | - |
| Zhang X (2016) | 613 | 236 | Down regulated | - | 0.45 (0.11, 0.60) | - |
| Prahm (2018) | 802 | 197 | Down regulated | - | - | 0.48 (0.29, 0.80) |
| Bagnoli (2016) | 890 | 179 | Down regulated | - | 0.09 (0.01, 0.72) | - |
| Minareci (2024) | 1181 | 69 | Up-regulated | - | 5.40 (2.00, 14.90) | 3.20 (1.60, 6.20) |
| Prahm (2018) | 1183 | 197 | Up-regulated | - | 1.42 (1.17, 1.74) | - |
| Flores-Colon (2024) | 1206 | 486 | Up-regulated | - | 1.78 (1.42, 2.25) | - |
| Xie (2020) | 1231 | 116 | Down regulated | - | 0.41 (0.21, 0.68) | - |
| Guo (2018) | 1294 | 69 | Down regulated | - | 0.38 (0.24, 0.60) | - |
| Minareci (2024) | 4314 | 69 | Up-regulated | - | 2.78 (1.30, 6.00) | 2.30 (1.30, 4.10) |
| Bagnoli (2016) | 100-3p | 179 | Down regulated | miR-99 family | 1.96 (1.29, 2.97) | - |
| Calura (2016) | 106b-5p | 157 | Up-regulated | miR-106 family | 4.35 (1.96, 10.00) | 3.45 (1.75, 6.67) |
| Flores-Colon (2024) | 10a | 486 | Up-regulated | miR-10 family | 1.31 (1.04, 1.65) | - |
| Calura (2016) | 125b-5p | 157 | Up-regulated | miR-125 family | 7.14 (2.38, 20.41) | 11.11 (4.76, 33.33) |
| Prahm (2018) | 126-3p | 197 | Up-regulated | miR-126 family | 1.38 (1.11, 1.71) | - |
| Bagnoli (2016) | 135b-5p | 179 | Up-regulated | - | 0.85 (0.76, 0.96) | - |
| Bagnoli (2016) | 141-3p | 179 | Up-regulated | miR-200 family | 0.82 (0.73, 0.92) | - |
| Calura (2016) | 141-3p | 157 | Up-regulated | miR-200 family | 0.33 (0.15, 0.73) | 0.35 (0.18, 0.68) |
| Bagnoli (2016) | 143-5p | 179 | Down regulated | - | 1.67 (1.18, 2.37) | - |
| Calura (2016) | 145-5p | 157 | Up-regulated | miR-143/145 family | 16.67 (4.17, 100.00) | 14.29 (5.00, 50.00) |
| Bagnoli (2016) | 151a-3p | 179 | Up-regulated | - | 1.36 (1.03, 1.51) | - |
| Calura (2016) | 15a-5p | 157 | Down regulated | miR-15/16 family | 0.21 (0.09, 0.48) | 0.25 (0.13, 0.50) |
| Calura (2016) | 16-5p | 157 | Down regulated | miR-15/16 family | 0.22 (0.09, 0.51) | 0.15 (0.07, 0.32) |
| Calura (2016) | 17-5p | 157 | Down regulated | miR-17-92 family | 5.88 (0.78, 50.00) | 4.35 (0.99, 20.00) |
| Panoutsopoulou (2020) | 181a | 81 | Up-regulated | miR-181 family | 2.20 (1.12, 4.31) | 2.39 (1.27, 4.48) |
| Petrillo (2016) | 181a-5p | 82 | Up-regulated | miR-181 family | 4.37 (2.63, 7.83) | 4.54 (2.63, 7.83) |
| Petrillo (2016) | 181b-5p | 82 | Up-regulated | miR-181 family | 1.11 (0.61, 1.99) | 1.10 (0.64, 1.66) |
| Lee (2012) | 181d | 68 | Up-regulated | miR-181 family | 0.42 (0.14, 1.23) | 0.34 (0.12, 0.94) |
| Petrillo (2016) | 181d | 82 | Up-regulated | miR-181 family | 0.85 (0.48, 1.53) | 0.84 (0.52, 1.34) |
| Bagnoli (2016) | 193a-5p | 179 | Up-regulated | miR-193 family | 1.98 (1.49, 2.61) | - |
| Li (2015) | 193b | 116 | Up-regulated | miR-193 family | 0.24 (0.12, 0.55) | - |
| Bagnoli (2016) | 193b-5p | 179 | Up-regulated | miR-193 family | 1.51 (1.06, 2.14) | - |
| Bagnoli (2016) | 195-3p | 179 | Down regulated | miR-15/16 family | 1.63 (1.13, 2.36) | - |
| Calura (2016) | 195-5p | 157 | Down regulated | miR-15/16 family | 0.13 (0.05, 0.36) | 0.05 (0.01, 0.20) |
| Pal (2024) | 199a | 208 | Up-regulated | miR-199 family | 0.77 (0.20, 3.02) | - |
| Marchini (2011) | 199a-3p | 89 | Up-regulated | miR-199 family | 10.36 (1.06, 101.57) | 6.43 (1.46, 28.29) |
| Petrillo (2016) | 199a-3p | 82 | Up-regulated | miR-199 family | 2.91 (1.60, 5.28) | 1.92 (1.19, 3.10) |
| Marchini (2011) | 199a-5p | 89 | Up-regulated | miR-199 family | 8.20 (0.88, 76.42) | 8.82 (1.14, 68.56) |
| Petrillo (2016) | 199a-5p | 82 | Up-regulated | miR-199 family | 1.98 (1.10, 3.56) | 1.62 (1.10, 2.61) |
| Petrillo (2016) | 199b-5p | 82 | Up-regulated | miR-199 family | 0.51 (0.27, 0.92) | 0.68 (0.41, 1.11) |
| Calura (2016) | 19a-3p | 157 | Down regulated | miR-17-92 family | 0.32 (0.12, 0.85) | 1.79 (0.68, 4.76) |
| Cao Q (2014) | 200a | 100 | Up-regulated | miR-200 family | 0.06 (0.03, 0.74) | - |
| Hu (2009) | 200a | 55 | Up-regulated | miR-200 family | 0.22 (0.09, 0.53) | - |
| Leskela (2011) | 200a | 72 | Up-regulated | miR-200 family | - | 0.82 (0.39, 1.75) |
| Meng (2016) | 200a | 163 | Up-regulated | miR-200 family | 1.70 (0.80, 3.50) | 1.10 (0.60, 1.90) |
| Bagnoli (2016) | 200a-3p | 179 | Up-regulated | miR-200 family | 0.81 (0.71, 0.93) | - |
| Cao Q (2014) | 200b | 100 | Up-regulated | miR-200 family | 0.06 (0.03, 0.88) | - |
| Kapetanakis (2015) | 200b | 51 | Up-regulated | miR-200 family | - | 2.95 (0.94, 9.28) |
| Leskela (2011) | 200b | 72 | Up-regulated | miR-200 family | - | 0.74 (0.34, 1.62) |
| Marchini (2011) | 200b | 89 | Up-regulated | miR-200 family | 2.05 (0.64, 6.57) | 2.34 (0.86, 6.36) |
| Meng (2016) | 200b | 163 | Up-regulated | miR-200 family | 2.70 (1.30, 5.70) | 1.60 (0.90, 2.80) |
| Bagnoli (2016) | 200b-3p | 179 | Up-regulated | miR-200 family | 0.79 (0.68, 0.91) | - |
| Cao Q (2014) | 200c | 100 | Up-regulated | miR-200 family | 0.06 (0.03, 0.79) | - |
| Elgaaen (2014) | 200c | 35 | Up-regulated | miR-200 family | 1.33 (1.04, 1.70) | 2.24 (1.08, 4.66) |
| Flores-Colon (2024) | 200c | 486 | Up-regulated | miR-200 family | 1.15 (0.92, 1.44) | - |
| Gao (2015) | 200c | 93 | Up-regulated | miR-200 family | 0.55 (0.30, 1.00) | - |
| Leskela (2011) | 200c | 72 | Up-regulated | miR-200 family | 0.48 (0.24, 0.97) | 0.45 (0.20, 1.00) |
| Marchini (2011) | 200c | 89 | Up-regulated | miR-200 family | 0.24 (0.08, 0.79) | 0.42 (0.15, 1.20) |
| Meng (2016) | 200c | 163 | Up-regulated | miR-200 family | 2.40 (1.20, 4.90) | 2.00 (1.10, 3.60) |
| Bagnoli (2016) | 200c-3p | 179 | Up-regulated | miR-200 family | 0.79 (0.69, 0.91) | - |
| Calura (2016) | 200c-3p | 157 | Up-regulated | miR-200 family | 0.18 (0.07, 0.49) | 0.24 (0.12, 0.49) |
| Flores-Colon (2024) | 203a | 486 | Up-regulated | miR-203 family | 1.18 (0.93, 1.49) | - |
| Calura (2016) | 20a-5p | 157 | Down regulated | miR-17-92 family | 0.17 (0.07, 0.46) | 0.35 (0.18, 0.67) |
| Calura (2016) | 20b-5p | 157 | Down regulated | miR-17-92 family | 0.18 (0.02, 1.33) | - |
| Prahm (2018) | 23a-3p | 197 | Up-regulated | miR-23 family | - | 1.70 (1.15, 2.51) |
| Bagnoli (2016) | 23a-5p | 179 | Up-regulated | miR-23 family | 1.64 (1.18, 2.28) | - |
| Prahm (2018) | 23a-5p | 197 | Up-regulated | miR-23 family | - | 1.32 (1.09, 1.61) |
| Calura (2016) | 26a-5p | 157 | Down regulated | miR-26 family | 0.20 (0.08, 0.52) | 0.14 (0.06, 0.35) |
| Pal (2024) | 27a | 208 | Up-regulated | miR-27 family | 1.09 (0.46, 2.60) | - |
| Calura (2016) | 29a-3p | 157 | Up-regulated | miR-29 family | 0.18 (0.08, 0.41) | 0.19 (0.07, 0.46) |
| Petrillo (2016) | 29a-3p | 82 | Up-regulated | miR-29 family | 1.00 (0.56, 1.74) | 1.20 (0.74, 1.92) |
| Bagnoli (2016) | 29a-5p | 179 | Up-regulated | miR-29 family | 1.77 (1.15, 2.70) | - |
| Flavin (2009) | 29b | 50 | Up-regulated | miR-29 family | 2.20 (1.03, 5.11) | - |
| Calura (2016) | 29b-3p | 157 | Up-regulated | miR-29 family | 0.20 (0.09, 0.45) | 0.20 (0.10, 0.39) |
| Petrillo (2016) | 29b-3p | 82 | Up-regulated | miR-29 family | 1.10 (0.61, 1.92) | 0.97 (0.60, 1.56) |
| Petrillo (2016) | 29c-3p | 82 | Up-regulated | miR-29 family | 1.41 (0.78, 2.51) | 1.42 (0.89, 2.27) |
| Bagnoli (2016) | 29c-5p | 179 | Up-regulated | miR-29 family | 1.60 (1.23, 2.07) | - |
| Marchini (2011) | 30a | 89 | Up-regulated | miR-30 family | 0.50 (0.11, 2.23) | 0.93 (0.31, 2.80) |
| Sestito (2015) | 30a | 39 | Up-regulated | miR-30 family | - | 0.84 (0.72, 0.98) |
| Wang (2013) | 30a | 69 | Up-regulated | miR-30 family | 0.18 (0.06, 0.53) | - |
| Zhao (2013) | 30a | 30 | Up-regulated | miR-30 family | 0.34 (0.13, 0.87) | - |
| Lee (2012) | 30a-3p | 68 | Up-regulated | miR-30 family | 0.48 (0.19, 1.18) | 0.46 (0.21, 1.00) |
| Petrillo (2016) | 30a-5p | 82 | Up-regulated | miR-30 family | 0.62 (0.34, 1.13) | 0.85 (0.53, 1.35) |
| Bagnoli (2016) | 30b-3p | 179 | Up-regulated | miR-30 family | 1.98 (1.24, 3.17) | - |
| Petrillo (2016) | 30b-5p | 82 | Up-regulated | miR-30 family | 1.33 (0.74, 2.38) | 1.10 (0.69, 1.75) |
| Lee (2012) | 30c | 68 | Up-regulated | miR-30 family | 0.37 (0.10, 1.44) | 0.27 (0.08, 0.92) |
| Petrillo (2016) | 30c-5p | 82 | Up-regulated | miR-30 family | 0.83 (0.46, 1.48) | 0.88 (0.54, 1.41) |
| Lee (2012) | 30d | 68 | Up-regulated | miR-30 family | 0.25 (0.07, 0.82) | 0.33 (0.12, 0.90) |
| Marchini (2011) | 30d | 89 | Up-regulated | miR-30 family | 0.64 (0.19, 2.18) | 0.60 (0.20, 1.78) |
| Bagnoli (2016) | 30d-5p | 179 | Up-regulated | miR-30 family | 1.25 (1.03, 1.52) | - |
| Petrillo (2016) | 30d-5p | 82 | Up-regulated | miR-30 family | 1.10 (0.58, 1.86) | 1.00 (0.65, 1.69) |
| Wang (2013) | 30e | 69 | Up-regulated | miR-30 family | 0.14 (0.04, 0.53) | - |
| Zhao (2013) | 30e | 30 | Up-regulated | miR-30 family | 0.20 (0.05, 0.85) | - |
| Lee (2012) | 30e-3p | 68 | Up-regulated | miR-30 family | 0.48 (0.17, 1.37) | 0.39 (0.16, 0.94) |
| Petrillo (2016) | 30e-5p | 82 | Up-regulated | miR-30 family | 0.98 (0.55, 1.76) | 1.10 (0.63, 1.62) |
| Calura (2016) | 324-5p | 157 | Up-regulated | miR-324 family | 0.73 (0.33, 1.64) | 1.45 (0.75, 2.78) |
| Bagnoli (2016) | 330-3p | 179 | Down regulated | - | 1.86 (1.21, 2.85) | - |
| Schmid (2016) | 34a | 133 | Down regulated | miR-34 family | 0.51 (0.29, 0.88) | 0.55 (0.31, 0.97) |
| Welponer (2020) | 34a | 228 | Down regulated | miR-34 family | 1.25 (0.83, 1.67) | 1.67 (1.00, 2.50) |
| Calura (2016) | 34a-5p | 157 | Down regulated | miR-34 family | 0.12 (0.06, 0.27) | 0.07 (0.03, 0.16) |
| Welponer (2020) | 34b | 228 | Down regulated | miR-34 family | 2.50 (1.25, 5.00) | 5.00 (2.00, 10.00) |
| Calura (2016) | 34b-5p | 157 | Down regulated | miR-34 family | 0.22 (0.10, 0.49) | 0.35 (0.18, 0.68) |
| Welponer (2020) | 34c | 228 | Down regulated | miR-34 family | 1.67 (1.00, 3.33) | 3.33 (1.43, 5.00) |
| Bagnoli (2016) | 423-5p | 179 | Up-regulated | - | 1.77 (1.23, 2.54) | - |
| Calura (2016) | 424-5p | 157 | Down regulated | - | 0.47 (0.19, 1.12) | 0.52 (0.26, 1.08) |
| Calura (2016) | 449a | 157 | Down regulated | miR-449 family | 0.40 (0.15, 1.09) | 0.36 (0.16, 0.81) |
| Bagnoli (2016) | 453-5p | 179 | Up-regulated | - | 1.28 (1.06, 1.53) | - |
| Bagnoli (2016) | 486-5p | 179 | Down regulated | - | 1.35 (1.12, 1.61) | - |
| Lee (2012) | 493-5p | 68 | Down regulated | - | 1.51 (0.57, 4.00) | 1.70 (0.74, 3.94) |
| Bagnoli (2016) | 506-3p | 179 | Down regulated | - | 0.64 (0.48, 0.84) | - |
| Bagnoli (2016) | 508-3p | 179 | Down regulated | - | 0.75 (0.64, 0.87) | - |
| Bagnoli (2016) | 509-3p | 179 | Down regulated | miR-509 family | 0.78 (0.69, 0.90) | - |
| Bagnoli (2016) | 509-5p | 179 | Down regulated | miR-509 family | 0.68 (0.56, 0.84) | - |
| Bagnoli (2016) | 513a-5p | 179 | Down regulated | miR-513 family | 0.74 (0.66, 0.89) | - |
| Bagnoli (2016) | 513b-5p | 179 | Down regulated | miR-513 family | 0.82 (0.73, 0.91) | - |
| Bagnoli (2016) | 514a-3p | 179 | Down regulated | - | 0.81 (0.73, 0.91) | - |
| Lee (2012) | 532-5p | 68 | Down regulated | miR-532 family | 0.84 (0.30, 2.36) | 0.69 (0.27, 1.74) |
| Wei (2018) | 532-5p | 145 | Down regulated | miR-532 family | 0.26 (0.21, 0.8) | - |
| Bagnoli (2016) | 574-5p | 179 | Up-regulated | - | 1.28 (1.05, 1.57) | - |
| Bagnoli (2016) | 769-5p | 179 | Up-regulated | - | 1.76 (1.19, 2.61) | - |
| Bagnoli (2016) | 99b-5p | 179 | Down regulated | miR-99 family | 1.35 (1.08, 1.70) | - |
| Lu (2011) | let-7a | 211 | Down regulated | let-7 family | 0.94 (0.57, 1.55) | 1.03 (0.62, 1.70) |
| Calura (2016) | let-7a-5p | 157 | Down regulated | let-7 family | 0.14 (0.04, 0.42) | 0.23 (0.10, 0.55) |
| Petrillo (2016) | let-7a-5p | 82 | Down regulated | let-7 family | 1.61 (0.90, 2.88) | 1.83 (1.13, 2.95) |
| Calura (2016) | let-7b-5p | 157 | Down regulated | let-7 family | 0.29 (0.13, 0.66) | 0.38 (0.16, 0.92) |
| Petrillo (2016) | let-7b-5p | 82 | Down regulated | let-7 family | 1.10 (0.59, 1.89) | 1.04 (0.65, 1.68) |
| Petrillo (2016) | let-7c | 82 | Down regulated | let-7 family | 1.10 (0.58, 1.90) | 0.89 (0.54, 1.44) |
| Petrillo (2016) | let-7d-5p | 82 | Down regulated | let-7 family | 1.48 (0.82, 1.66) | 1.43 (0.89, 2.30) |
| Calura (2016) | let-7e-5p | 157 | Down regulated | let-7 family | 0.04 (0.01, 0.12) | 0.02 (0.01, 0.07) |
| Petrillo (2016) | let-7e-5p | 82 | Down regulated | let-7 family | 1.18 (0.66, 2.12) | 1.10 (0.63, 1.65) |
| Zheng (2013) | let-7f | 360 | Down regulated | let-7 family | 0.93 (0.56, 1.52) | 0.48 (0.30, 0.79) |
| Petrillo (2016) | let-7f-5p | 82 | Down regulated | let-7 family | 0.76 (0.42, 1.38) | 0.79 (0.49, 1.27) |
| Petrillo (2016) | let-7g-5p | 82 | Down regulated | let-7 family | 1.82 (1.10, 3.32) | 1.84 (1.12, 2.99) |
| Petrillo (2016) | let-7i-5p | 82 | Down regulated | let-7 family | 0.78 (0.43, 1.41) | 0.92 (0.57, 1.49) |

Note: HR, hazard ratio; OS, overall survival; PFS, progression-free survival

**Table S6. Association between each miRNA family and the prognosis of ovarian cancer.**

| Category | Number of studies | Hazard Ratio | *I^2^* |
| --- | --- | --- | --- |
| **Up-regulated microRNA** |  |  |  |
| **Overall survival** |  |  |  |
| miR-181 | 3 | 1.15 (0.48, 1.83) | 68% |
| miR-199 | 3 | 1.32 (0.35, 2.29) | 55% |
| miR-200 | 12 | **0.66 (0.53, 0.80)** | 87% |
| miR-29 | 4 | 1.01 (0.55, 1.47) | 90% |
| miR-30 | 6 | **0.63 (0.39, 0.87)** | 79% |
| miR-96 | 3 | 1.20 (0.81, 1.58) | 69% |
| **Progression-free survival** |  |  |  |
| miR-181 | 3 | 1.12 (0.48, 1.76) | 77% |
| miR-200 | 8 | **0.68 (0.47, 0.89)** | 66% |
| miR-30 | 4 | **0.70 (0.54, 0.87)** | 45% |
| **Down-regulated microRNA** |  |  |  |
| **Overall survival** |  |  |  |
| let-7 | 4 | 0.83 (0.53, 1.14) | 90% |
| miR-34 | 3 | 0.64 (0.27, 1.01) | 88% |
| **Progression-free survival** |  |  |  |
| let-7 | 4 | 0.83 (0.53, 1.13) | 91% |

**Table S7. Subgroup analysis of microRNA-200 family and microRNA-30 family.**

| Category | Number of studies | HR (95%CI) | *I^2^* | P value |
| --- | --- | --- | --- | --- |
| **MicroRNA-200 family** |  |  |  |  |
| Country |  |  |  | 0.003 |
| China | 3 | 0.32 (0.06, 0.58) | 66% |  |
| Other region | 9 | 0.77 (0.63, 0.91) | 86% |  |
| Sample source |  |  |  | 0.133 |
| tissue | 8 | 0.65 (0.50, 0.79) | 89% |  |
| blood | 2 | 1.11 (0.52, 1.69) | 78% |  |
| **MicroRNA-30 family** |  |  |  |  |
| Sample source |  |  |  | 0.011 |
| tissue | 2 | 1.16 (0.65, 1.67) | 46% |  |
| FFPE | 4 | 0.46 (0.29, 0.64) | 54% |  |

Note: HR, hazard ratio

**Table S8. Egger’s test value for each microRNA.**

| **Type of microRNA** | **P-value for Egger** |
| --- | --- |
| **Overall survival** |  |
| MicroRNA-200 family | 0.350 |
| MicroRNA-30 family | 0.001 |
| MicroRNA-34 family | 0.311 |
| let-7 family | <0.001 |
| **Progression-free survival** |  |
| MicroRNA-200 family | 0.895 |
| MicroRNA-30 family | 0.108 |
| MicroRNA-34 family | 0.543 |
| let-7 family | 0.002 |

**Supplementary figure legends**

**Figure S1.** Subgroup analysis of microRNA-200 family by their location in chromosome.

**Figure S2.** Sensitivity analysis of meta-analysis of miRNA-200 family for overall survival (A) and progression-free survival (B) of ovarian cancer.

**Figure S3.** Funnel plot of meta-analysis of miRNA-200 family for overall survival (A) and progression-free survival (B) of ovarian cancer.

**Figure S4.** Sensitivity analysis of meta-analysis of miRNA-30 family for overall survival (A) and progression-free survival (B) of ovarian cancer.

**Figure S5.** Funnel plot and trim and fill analyses of meta-analysis of miRNA-30 family for prognosis of ovarian cancer. (A) Overall survival, (B) Progression-free survival, (C) trim and fill analysis funnel plot (8 studies added compared with initial meta-analysis of miRNA-30 family for OS of ovarian cancer)


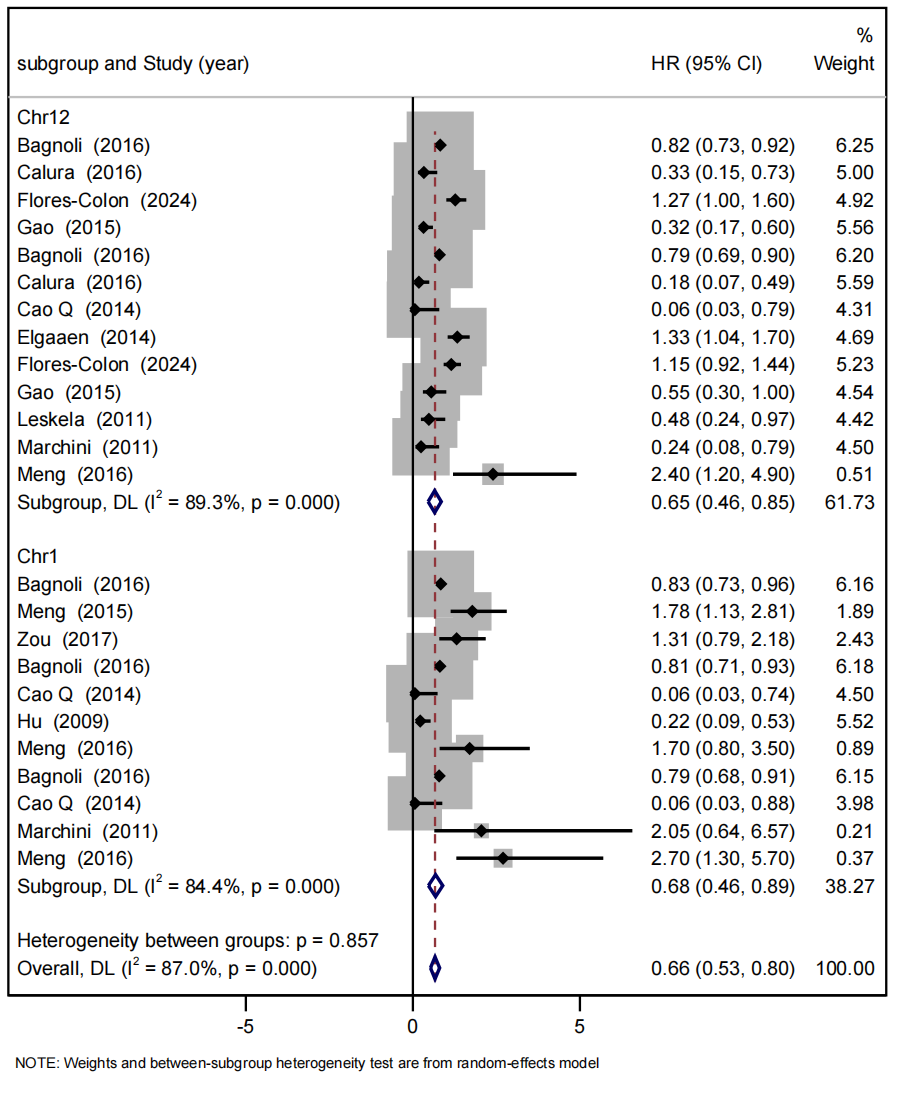


**Figure S1. Subgroup analysis of microRNA-200 family by their location in chromosome.**


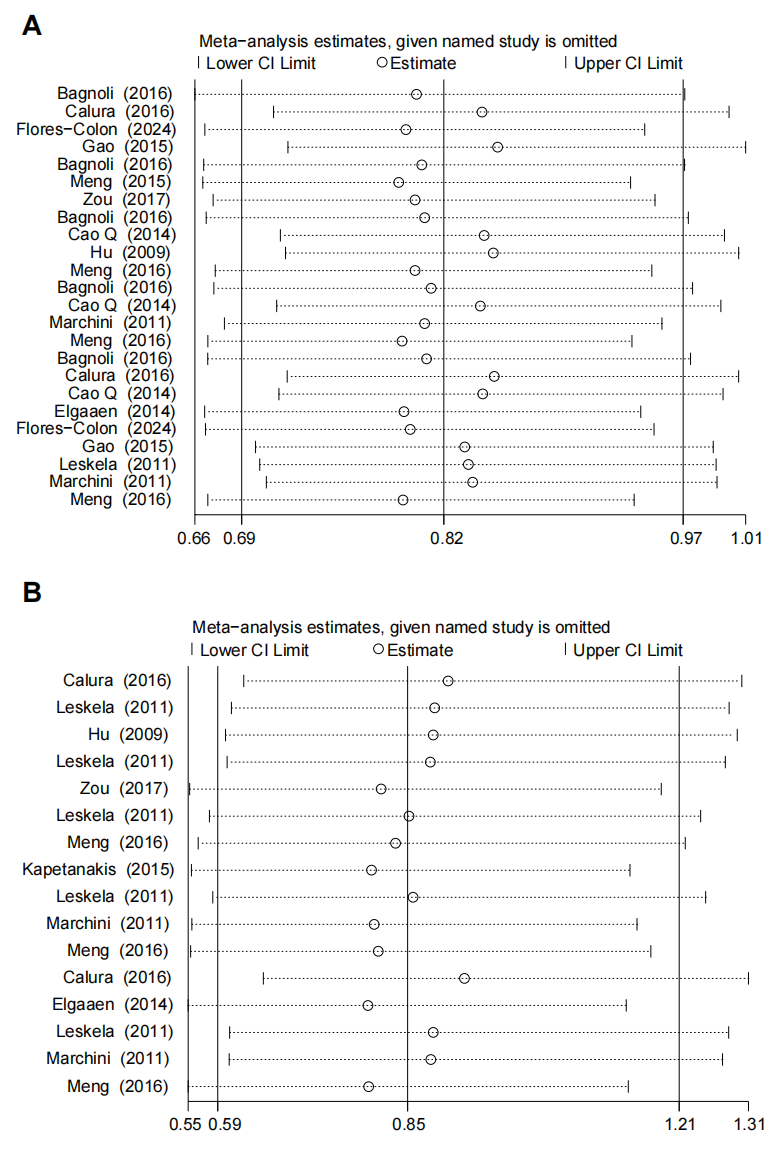


**Figure S2. Sensitivity analysis of meta-analysis of miRNA-200 family for overall survival (A) and progression-free survival (B) of ovarian cancer.**


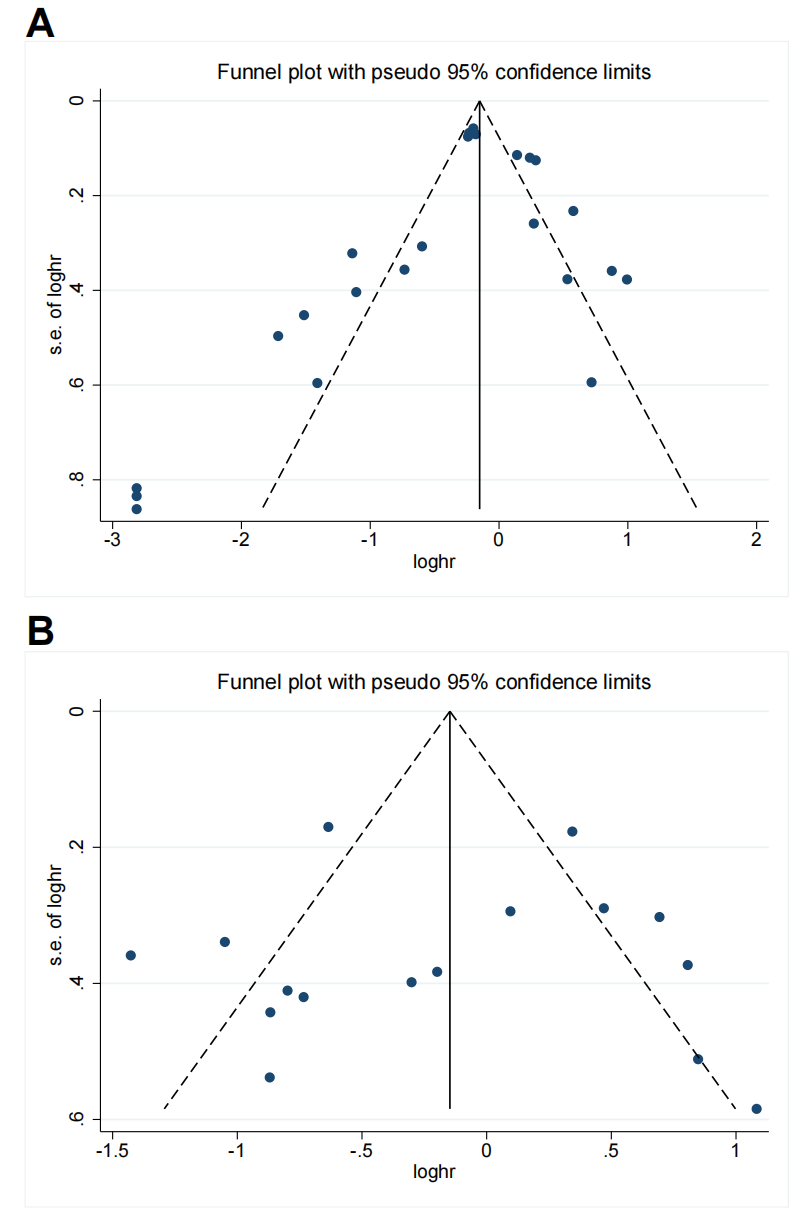


**Figure S3. Funnel plot of meta-analysis of miRNA-200 family for overall survival (A) and progression-free survival (B) of ovarian cancer.**


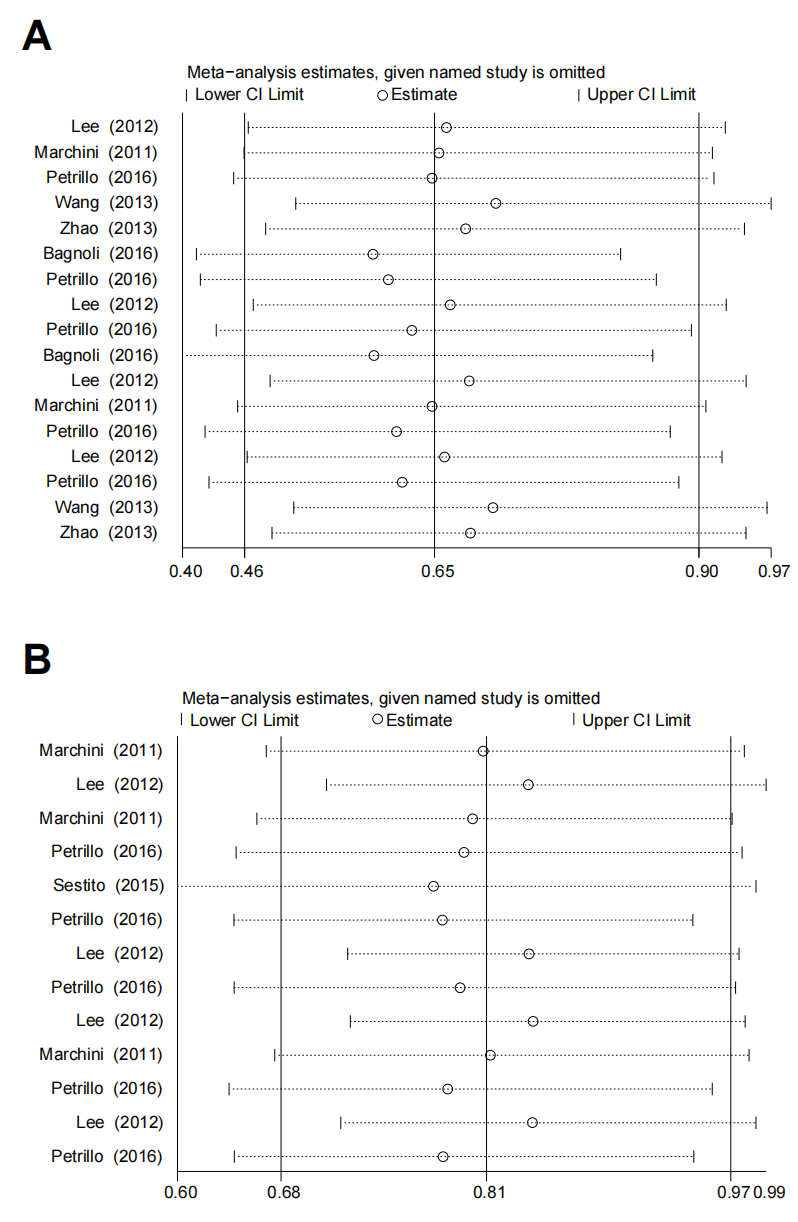


**Figure S4. Sensitivity analysis of meta-analysis of miRNA-30 family for overall survival (A) and progression-free survival (B) of ovarian cancer.**


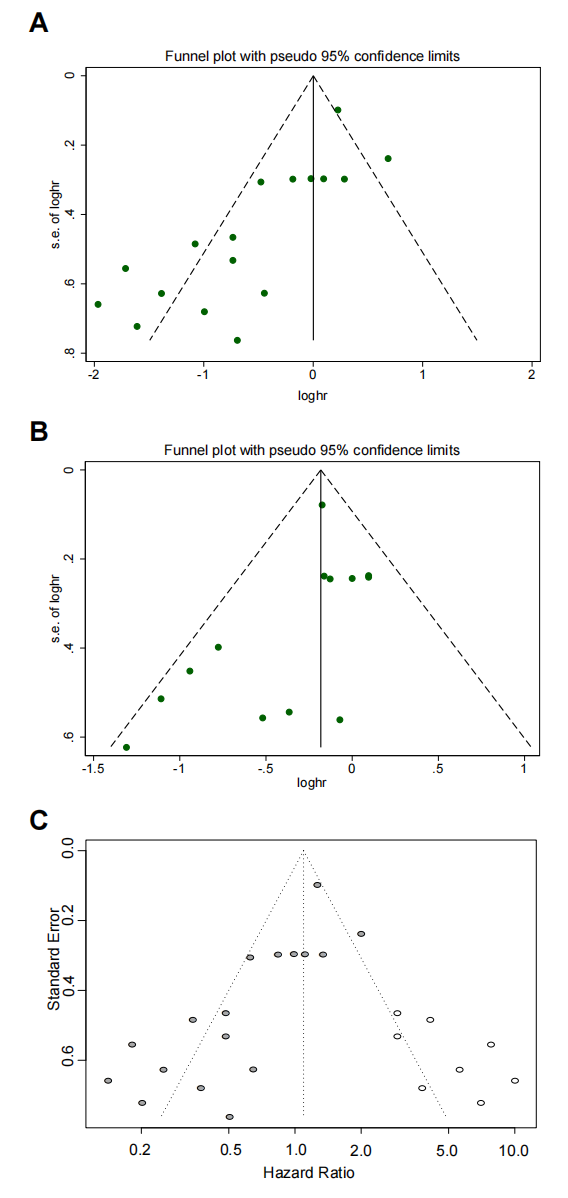


**Figure S5. Funnel plot and trim and fill analyses of meta-analysis of miRNA-30 family for prognosis of ovarian cancer. (A) Overall survival, (B) Progression-free survival, (C) trim and fill analysis funnel plot (8 studies added compared with initial meta-analysis of miRNA-30 family for OS of ovarian cancer)**
